# CIC protein instability contributes to tumorigenesis in glioblastoma

**DOI:** 10.1038/s41467-018-08087-9

**Published:** 2019-02-08

**Authors:** Severa Bunda, Pardeep Heir, Julie Metcalf, Annie Si Cong Li, Sameer Agnihotri, Stefan Pusch, Mamatjan Yasin, Mira Li, Kelly Burrell, Sheila Mansouri, Olivia Singh, Mark Wilson, Amir Alamsahebpour, Romina Nejad, Bethany Choi, David Kim, Andreas von Deimling, Gelareh Zadeh, Kenneth Aldape

**Affiliations:** 1 MacFeeters Hamilton Centre for Neuro-Oncology Research, Princess Margaret Cancer Centre, Toronto, ON M5G 2C1 Canada; 2 Division of Neurosurgery, Toronto Western Hospital, Toronto, ON M5G 2C1 Canada; 3 Insititute of Medical Science, University Health Network and University of Toronto, Toronto, ON M5S 3E1 Canada; 4 Laboratory of Pathology, National Cancer Institute, Bethesda, MD 20892 USA; 5 Department of Neuropathology, Institute of Pathology, Heidelberg University Hospital, Heidelberg, D-69120 Germany; 6 Present Address: German Consortium of Translational Cancer Research (DKTK), Clinical Cooperation Unit Neuropathology German Cancer Research Center (DKFZ), Heidelberg, D-69120 Germany; 7 Department of Neurosurgery, University of Pittsburgh Medical Center, UPMC Presbyterian, Suite B-400, 200 Lothrop Street, Pittsburgh, PA 15213 USA

## Abstract

Capicua (CIC) is a transcriptional repressor that counteracts activation of genes downstream of receptor tyrosine kinase (RTK)/Ras/ERK signaling. It is well-established that tumorigenesis, especially in glioblastoma (GBM), is attributed to hyperactive RTK/Ras/ERK signaling. While CIC is mutated in other tumors, here we show that CIC has a tumor suppressive function in GBM through an alternative mechanism. We find that CIC protein levels are negligible in GBM due to continuous proteasome-mediated degradation, which is mediated by the E3 ligase PJA1 and show that this occurs through binding of CIC to its DNA target and phosphorylation on residue S173. PJA1 knockdown increased CIC stability and extended survival using in-vivo models of GBM. Deletion of the ERK binding site resulted in stabilization of CIC and increased therapeutic efficacy of ERK inhibition in GBM models. Our results provide a rationale to target CIC degradation in Ras/ERK-driven tumors, including GBM, to increase efficacy of ERK inhibitors.

## Abstract (web summary)

Capicua (CIC) is a tumour suppressor in oligodendroglioma. Here, the authors show that ERK activation mediates CIC regulation via ubiquitination and degradation by PJA1 and a degradation resistant form of CIC enhances efficacy of ERK inhibition in glioblastoma.

## Introduction

Glioblastoma (GBM) is the most common and malignant primary neuroepithelial tumor and remains incurable despite aggressive therapy. Molecular alterations of various signaling pathways potentiate receptor tyrosine kinase (RTK) activation, such as the frequent EGFR amplifications or variant III mutations (EGFRvIII) that are linked with the aggressive behavior of GBMs^1–3^. Unfortunately, results from clinical trials targeting Ras/Raf/MEK/ERK signaling downstream of RTK have only had limited success^4^, indicating a need for increasing understanding of the mechanisms regulating this pathway in GBM.

The high-mobility group (HMG)-box transcriptional repressor capicua (*CIC*) has recently emerged as a conserved nuclear sensor of RTK signaling in *Drosophila* and mammals^5^. In unstimulated cells, CIC represses EGFR/Ras pathway-responsive genes. Following EGFR stimulation, CIC repression is relieved, allowing for the expression of target genes. The best-characterized CIC targets in mammalian cells are the oncogenic transcription factors ETV1, ETV4, and ETV5^5^, which mediate cell proliferation, motility and invasion downstream of Ras^6^. Much has been learned from studies in *Drosophila*, where *CIC* was first described to be involved in developmental patterning and cell fate modulated through EGFR activation^7–10^, in a manner termed ‘default repression’. While CIC’s function is less well-understood in vertebrate organisms, the importance of CIC protein in maintaining cellular homeostasis downstream of EGFR/Ras/ERK signaling has recently become evident in humans^11–13^, but the molecular mechanisms governing CIC functions in normal cells and in cancer are lacking.

Investigation into the molecular function of CIC in cancer and GBM in particular, is further merited by recent findings connecting CIC’s downstream target ETV1 in GBM^14^.

*CIC* is not mutated in GBM, but mutations of this gene, located on chromosome 19q, occur in 70% of 1p19q-co-deleted oligodendrogliomas^15–18^. Decreased CIC expression is correlated with poorer outcome in these tumors^19^. Two CIC isoforms exist that differ in size, the short (CIC-S) and the long (CIC-L), and in their N-terminal region^20^. Given that the disease-associated mutations map to the CIC-S isoform of the protein, which suggests that the CIC-S isoform may be more important in tumorigenesis, we focus on the CIC-S isoform in the current study referred to as CIC throughout the manuscript^21^. In addition to loss-of-function mutations in oligodendrogliomas, and other tumor types, translocation events resulting in gene fusions of *CIC* with either *DUX4* or *FOXO4* has been shown in round cell sarcomas^22,23^. Additionally, CIC has most recently been shown to suppress invasion and metastasis in lung cancer, through an effector identified as MMP24^12^. In addition, germline CIC inactivation in adult mice was shown to induce T-cell acute lymphoblastic lymphoma^24^. Despite clear genetic evidence of its connection to one of the most important pathways in cancer, molecular mechanisms governing CIC regulation by Ras/ERK signaling and its potential involvement in GBM remain unknown.

In this study, we present data to establish a role for *CIC* in GBM. We find that activation of Ras/ERK signaling mediates ubiquitylation and degradation of CIC by a nuclear E3 ligase PRAJA1 (PJA1) to drive GBM growth. We provide mechanistic insights into regulation of CIC downstream of EGFR activation via serine/threonine phosphorylation. Importantly, a degradation-resistant CIC mutant, insensitive to the effects of ERK stimulation, resulted in suppression of GBM growth and sensitized tumors to the effects of ERK inhibition, a potential therapeutic opportunity for further study in this aggressive neoplasm.

## Results

### CIC protein levels are low in GBM despite robust mRNA levels

**Fig. 1:**
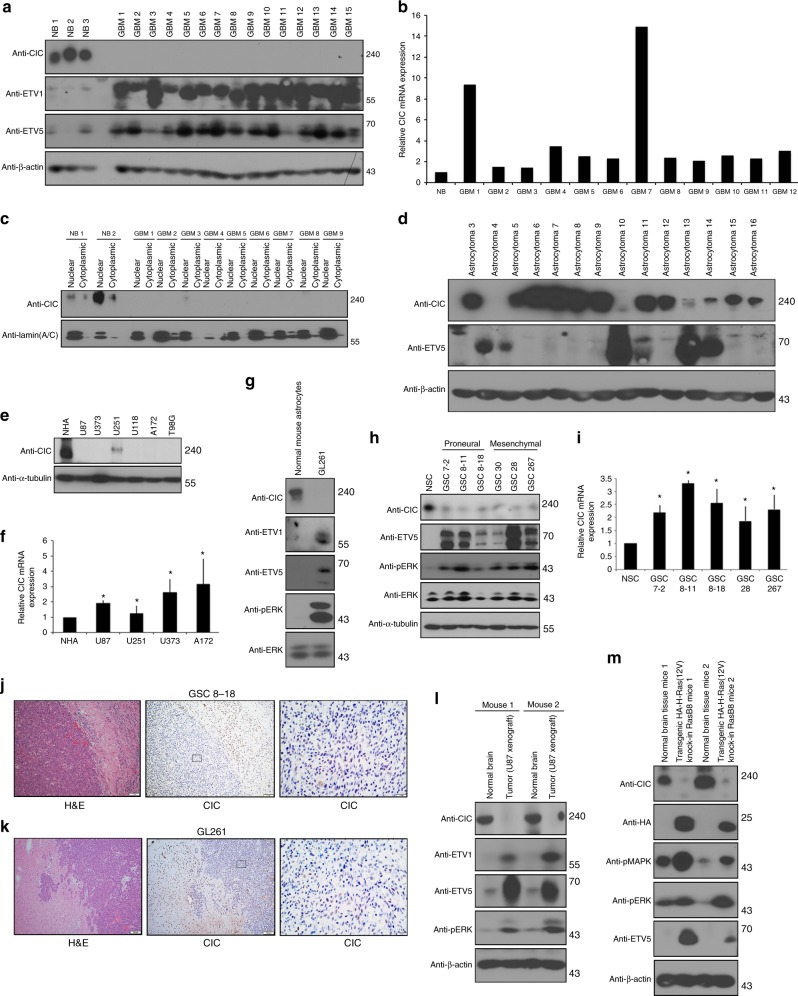
Expression of CIC and its targets in human GBM tumors and cells. Human operative GBM samples or normal derived brains (NB) were lysed and **a** immunoblotted with indicated antibodies **b** or total RNA extracted and quantitative real-time PCR analysis was carried out using TaqMan gene expression assays. The graph depicts fold changes in CIC expression relative to normal brain. **c** Nuclear or cytoplasmic lysates were isolated from human operative GBM samples or normal brain and were immunoblotted with indicated antibodies. **d** Human operative astrocytoma samples were lysed and immunoblotted with indicated antibodies. Human-derived GBM cell lines or normal human astrocytes (NHA) were lysed and **e** protein lysates were immunoblotted with indicated antibodies (**f**) or total RNA extracted and quantitative real-time PCR analysis was carried out using TaqMan gene expression assays. The graph depicts fold changes in CIC expression relative to NHA. Data represent mean ± s.e.m. of three independent experiments performed in triplicate. *P < 0.05 Student’s *t*-test compared with NHA. **g** GL261 cells or normal mouse astrocytes were lysed and protein lysates were immunoblotted with indicated antibodies. Glioma stem cells (GSCs) were lysed and **h** protein lysates were immunoblotted with indicated antibodies (**i**) or total RNA extracted and quantitative real-time PCR analysis was carried out using TaqMan gene expression assays. The graph depicts fold changes in CIC expression relative to normal neural stem cells (NSC). Data represent mean ± s.e.m. of three independent experiments performed in triplicate. *P < 0.05 Student’s *t*-test compared with NSC. Representative images of hematoxylin and eosin (H&E) staining and immunohistochemistry using anti-CIC antibody of sections obtained from brains of intracranial xenograft (**j**) (GSC (8-18)) scale bar, 1 mm, or **k** GL261 mice scale bar, 500 μm. **l** Tumors or unaffected normal brains obtained from intracranial U87 xenograft were lysed and protein lysates were immunoblotted with indicated antibodies. **m** High-grade tumors obtained from two different oncogenic HA-H-Ras(12 V) knock-in RasB8 transgenic mice or from two different normal tissue obtained from wild-type background mice were lysed and protein lysates were immunoblotted with indicated antibodies. The immunoblot data are representative of at least three separate experiments

Information is lacking regarding the mechanism by which Ras/ERK signaling regulates CIC to alleviate target gene repression. In particular, it is not established whether CIC is as an important signaling regulator in GBM. The ETS family of oncogenic transcription factors, ETV1, ETV4, and ETV5 downstream of RTK/Ras/ERK activation have been shown to mediate gliomagenesis^14,25^, yet the role of CIC, a well-established repressor of these genes^21^, is unknown. To explore this, we first examined the expression of CIC protein in human newly diagnosed GBM human tumors. Interestingly, in 30/30 GBM patient tumor samples, CIC protein level was substantially reduced or absent compared to lysates derived from non-neoplastic brain tissue (Figure 1a and Supplementary Figure 1A and C). By contrast, *CIC* mRNA expression was readily detected in these samples, at levels equal to or exceeding that of normal brain (Fig. 1b and Supplementary Figure 1B and D). Further investigation of the nuclear fraction and confirmed that CIC was localized to the nuclear fraction in normal brain, but was indeed absent nuclear fractions of GBM tumors (Figure 1c). Extending this to lower-grade gliomas, CIC protein expression was detected at much higher levels in the majority of lower-grade astrocytoma samples studied (Fig. 1d). The loss of CIC protein in patient-derived samples correlated with de-repression the CIC targets, ETV1 and ETV5, in human GBM (Figure 1a and Supplementary Figure 1C) and in lower-grade astrocytoma (Fig. 1d). In addition to these experimental findings, we mined Cancer Genome Atlas (TCGA) data, to show that ETV1, ETV5, and ETV4 mRNA levels were higher in human GBM samples relative to normal brain (Supplementary Figure 1F).

Extending this to in vitro model systems, we found, similar to patient-GBM tumor samples, that CIC protein levels were reduced in human-derived established GBM cell lines, despite abundant CIC mRNA levels (Fig. 1e, f). CIC level was also negligible in a well-established mouse glioma 261 (GL261) cell line that harbors mutations in the K-Ras and p53 genes, frequently used for GBM therapy^26^, compared to normal mouse astrocytes (Figure 1g). Consistently, GBM cell lines also showed elevated ETV1, ETV5, and ETV4 expression compared to normal human astrocytes (NHA) (Figure 1g, Supplementary Figure 1G and H). Likewise, loss of CIC protein and elevated ETV levels were also seen in six different patient-derived glioma stem cells (GSC) compared to normal human stem cells (NSC) (Figure 1h, i).

To establish in vivo relevance, we generated intracranial xenografts of GBMs using murine glioma GL261 cells, GSC model and an established cell line (GSC 8-18 and U87). All three models revealed that CIC protein remained low and were substantially reduced in intracranial xenografts compared to normal contralateral brain parenchyma. Consistently, the levels of phosphorylated ERK (pERK), ETV1, and ETV5 were high in tumors compared to normal unaffected brain (Fig. 1j–l and Supplementary Figure 1I). Additionally, we investigated CIC in Ras-driven transgenic mouse model of GBM, (RasB8)^27^. High-grade tumors from RasB8 mice were isolated and compared to wild-type control mice. In agreement with our xenograft model, RasB8 tumors displayed CIC protein loss and a concomitant increase in ETV1 and ETV5 (Figure 1m). Taken together, these results indicate that CIC is strikingly reduced or absent at the protein level in GBM, while mRNA remains high, together with de-repression of ETV downstream targets.

### Effects of CIC on proliferation and gene expression

**Fig. 2:**
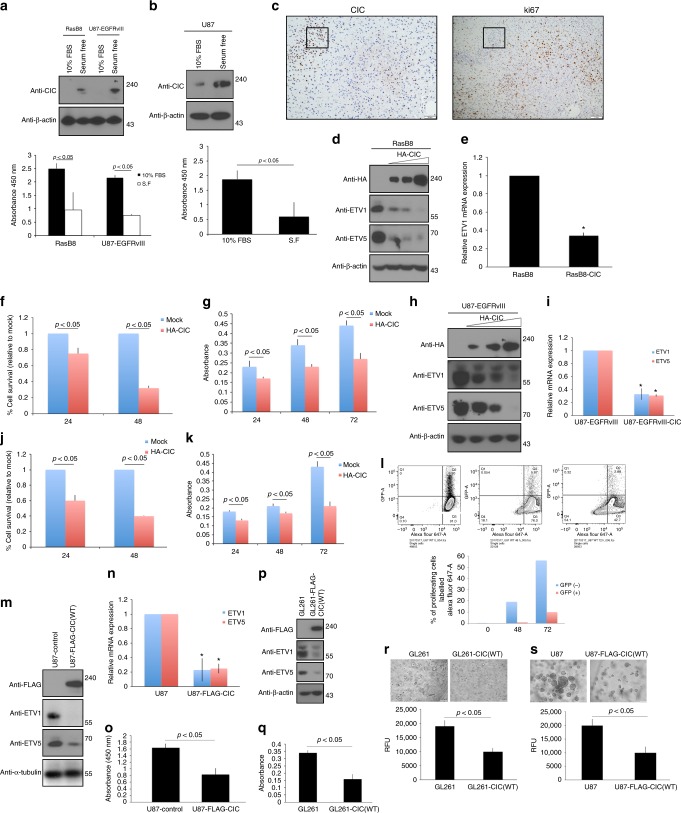
IC overexpression and cell proliferation. **a** RasB8, U87-EGFRvIII or **b** U87 were either serum-starved or maintained in 10% FBS, lysed and immunoblotted with indicated antibodies (top panel) or BrdU incorporation assay conducted (bottom panel). **c** Representative immunohistochemistry images using anti-Ki67 (scale bar, 500 μm) or anti-CIC (scale bar, 1 mm) antibody of high-grade tumors from RasB8 transgenic mice. **d** RasB8 transfected with or without increasing HA-CIC plasmid lysed and immunoblotted with indicated antibodies. **e** Quantitative real-time PCR analysis of RasB8 transfected with or without HA-CIC. The graph depicts fold changes in ETV1 expression relative to control. RasB8 transfected with or without HA-CIC plated equally plated and cell viability was assessed at indicated time points (in hours) by trypan blue exclusion or **f** alamar blue assay (**g**). **h** U87-EGFRvIII transfected with or without increasing concentrations of HA-CIC lysed and protein immunoblotted with indicated antibodies. **i** Quantitative real-time PCR analysis of U87-EGFRvIII transfected with or without HA-CIC. The graph depicts fold changes in ETV1 or -5 expression relative to control. U87-EGFRvIII transfected with or without HA-CIC plated equally and cell viability assessed at indicated time points (in hours) by trypan blue exclusion or **j** alamar blue assay **k**. **l** U87-EGFRvIII transfected with GFP-CIC labeled with eFluor 670 proliferation dye analyzed by flow cytometry. Graphs depict percentage of proliferating GFP-positive versus GFP-negative cells within the same population. Data are representative of at least three independent experiments. U87-Flag-CIC or control cells lysed and **m** protein lysates immunoblotted with indicated antibodies, **n** quantitative real-time PCR analysis conducted assessing ETV- or -5 expression or **o** BrdU incorporation assay. GL261-Flag-CIC or controls were lysed and **p** immunoblotted with indicated antibodies or **q** BrdU incorporation assay conducted. Anchorage-independent growth assay of GL261 (**r**) or U87 (**s**) cells expressing Flag-CIC showing fold changes relative to control (bottom panel) or phase contrast microscopy images (top panel). All graphs represent mean ± s.e.m. of three independent experiments performed either in octuplet for viability assays or in triplicate for real-time analysis. *P < 0.05 Student’s *t*-test compared with control. The immunoblot data are representative of at least three separate experiments

Using a panel of cultured cells, including serum-starved astrocytes derived from RasB8 mice as well as several GBM cell lines (including U87-EGFRvIII, with activated EGFR-ERK/Ras signaling) and HEK293 cells, we found that serum starvation and the consequent inhibition of cell proliferation correlated with increased CIC protein expression (Fig. 2a, b, Supplementary Figure 2A and B). This correlation was recapitulated in vivo in RasB8 tumors, showing CIC expression to be most prominent in areas where Ki67 expression was absent (Fig. 2c), and suggesting an inverse relationship between cell proliferation and CIC protein expression.

We then assessed effects of CIC overexpression. Ectopic expression of CIC attenuated Ras(G12D)-induced proliferation of HEK293 cells (Supplementary Figure 2D–E). Extending this to RasB8 astrocytes, U87, U87-EGFRvIII, GSC 8-18 and GL261 cells using alamar blue, trypan blue exclusion and bromodeoxyuridine (BrdU) incorporation assays the results collectively demonstrate that CIC expression, which correlates with reduced ETV’s protein and mRNA expression, significantly attenuated proliferation of these cells (Fig. 2d–q and Supplementary Figure 2E–L). Conversely, overexpression of ETV1, ETV4 or ETV5 in U87 and GL261 cells stably expressing CIC reversed the low proliferation rate of these cells (Supplementary Figure 2M and N). This is consistent with previous reports demonstrating the importance of ETVs in gliogenesis^25^. To examine CICs effect on proliferation in greater depth, we transfected GFP-CIC into U87- and U87-EGFRvIII cells that were subsequently labeled with eFluor 670 cell proliferation dye to monitor the division of individual cells. CIC-expressing cells underwent significantly fewer divisions compared to GFP-negative control cells within the same cell population (Fig. 2l and Supplementary Figure 2J). To further examine effects of CIC overexpression, we performed expression microarray analysis of *CIC*-transfected U87 and HEK293 cells versus controls and identified genes concordantly downregulated in both cell lines. Gene set enrichment analysis (GSEA) of gene sets that differed as a function of CIC expression identified oncogenic RTK/Ras/Raf/ERK pathways to be significantly downregulated (Supplementary Figure 2O), a finding that is consistent with CIC’s negative effect on proliferation. Importantly we show that CIC significantly reduces the transformation ability of U87 and GL261 parental cells by hampering their ability to grow in soft agar (Fig. 2r, s).

**Fig. 3:**
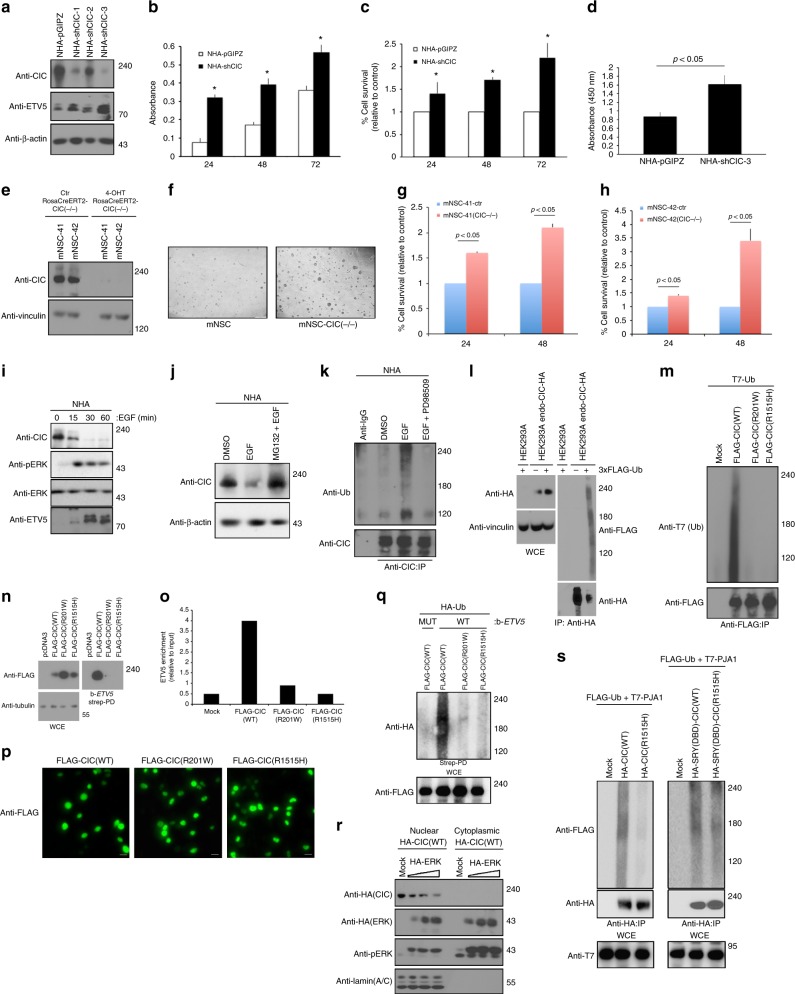
CIC knockdown, nuclear localization and ERK-mediated proteasomal degradation. **a** NHA-shCIC-1, 2 or 3 were lysed and immunoblotted with indicated antibodies. **b** Alamar blue, **c** trypan blue exclusion or **d** BrdU incorporation assay of NHA-shCIC-3 or control cells. mNSC-RosaCreERT2-CIC(−/−)-41 or (−/−)42 treated with or without 1 μM 4-OHT lysed and **e** immunoblotted with indicated antibodies or **f** imaged following 7 days in culture. Scale bar, 2 mm. Alamar blue assay of **g** mNSC-RosaCreERT2-CIC(−/−)-41 or **h** -42 cells treated with or without 1 μM 4-OHT. **i** NHA treated with or without EGF lysed and immunoblotted with indicated antibodies. **j** NHA pre-treated with MG132 or treated with or without EGF lysed and immunoblotted with indicated antibodies. **k** NHA pre-treated with MG132 prior to 1 h PD98509 pre-treatment, in the presence or absence of EGF lysed and IP with anti-CIC antibody or with anti-normal rabbit IgG and immunoblotted with indicated antibodies. **l** HEK293 endogenously tagged HA-CIC transfected with indicated plasmids pre-treated with MG132 prior to EGF treatment were lysed and a denaturing IP using anti-HA antibody was performed. **m** HEK293 transfected cells pre-treated with MG132 prior to EGF treatment were lysed and IP with anti-Flag antibody. **n** HEK293 transfected cells were lysed and incubated with Streptavidin agarose bound to biotinylated ETV5 oligonucleotides octameric motif followed by immunoblotting. **o** ChIP followed by quantitative PCR on the ETV5 promoter of HEK293 transfected cells. Graphs depict amount of ETV5 promoter enriched relative to input. **p** Immunofluorescence microscopy using anti-FLAG antibody of HEK293 cells transfected with indicated plasmids. Scale bar, 100 μm. **q** HEK293 transfected cells pre-treated with MG132 prior to EGF treatment lysed and Streptavidin agarose bound to either mutant (MUT) or wild type (WT) biotinylated ETV5 oligonucleotides octameric motif assay was performed. **r** Nuclear or cytoplasmic lysates of HEK293 cells transfected with increasing HA-ERK plasmid immunoblotted with indicated antibodies. **s** HEK293 transfected cells pre-treated with MG132 prior to EGF treatment were lysed IP with anti-HA antibody. IP Immunoprecipitated, strep-PD Streptavidin pull down, WCE whole-cell extract, Ub ubiquitin. Data represent mean ± s.e.m. of three independent experiments performed in octuplet. *P < 0.05 Student’s *t*-test compared with control

Since CIC expression reduced cell proliferation and transformation ability of GBM cells we asked whether *CIC* loss in normal cells enhanced their basal proliferation rate. Knockdown of CIC in NHA cells using lentiviral shRNA increased proliferation in a dose-dependent manner correlating with increased ETV mRNA expression and protein level (Fig. 3a–d and Supplementary Figure 3A). Similar findings were observed in *CIC*-null HEK293 cells generated with two different single guide (sg) RNAs (Supplementary Figure 3B and C). We also deleted *CIC* using (Z)-4-Hydroxytamoxifen treatment in mouse neural stem cells (mNSC) conditional for CIC expression in a RosaCreERT2 background (mNSC-RosaCreERT2-CIC(−/−)-41 and -42). Here, loss of *CIC* correlated with increased proliferation, increased numbers of neurospheres and enhanced neurosphere size (Fig. 3e–h, Supplementary Figure 3D and E).

Since loss-of-function mutations in *CIC* occur in a subset of IDH-mutant, 1p19q co-deleted oligodendrogliomas, we examined genes that were differentially expressed in *CIC* mutant versus *CIC* wild-type tumors using TCGA oligodendroglioma data. RNAseq data from TCGA identified 221 differentially expressed genes in *CIC* mutant versus wild-type oligodendrogliomas (Figure S3F, fold cutoff of 1.5, *p*-value < 0.05 and FDR < 10%) amongst which the known CIC targets ETV1, ETV4, and ETV5 were significantly up-regulated in *CIC*-mutant cases. GSEA identified several oncogenic pathways significantly enriched in the CIC-mutant oligodendrogliomas (Figure S3G) of which K-Ras signaling was significantly upregulated (Supplementary Figure 3H), corroborating the observation that CIC antagonizes Ras signaling and proliferation in glioma.

### Nuclear CIC is ubiquitylated and degraded in response to ERK

In an effort to elucidate the underlying mechanism of CIC protein loss in GBM, we first tested the effects of EGFR activation on CIC protein levels in non-neoplastic cells. We treated NHA and HEK293 cells with EGF (Fig. 3i, j and Supplementary Figure 3I-K), and found that short-term (30–60 min) EGF stimulation resulted in reduced CIC levels while concomitantly increasing the level of ETV5 (Fig. 3i). Importantly, 18 h after EGF treatment (following termination of active signaling), CIC protein was restored to basal levels (Supplementary Figure 3I and J). We hypothesized that the initial turnover of CIC in response to EGF was caused by ubiquitin-mediated proteasomal degradation. Consistent with this hypothesis, treatment with the proteasomal inhibitor MG132 prevented CIC reduction in response to EGF (Fig. 3j). Furthermore, treatment of NHA and HEK293 cells with EGF and MG132 promoted the accumulation of endogenous ubiquitylated CIC (Fig. 3k, l). To test the role of ERK activation in this process we used PD98509, an ERK inhibitor. We found that the accumulation of ubiquitylated CIC was eliminated by short-term ERK inhibition (1 h) (Fig. 3k). Conversely, in MG132-treated HEK293 cells CIC was robustly ubiquitylated only in the presence of ERK (Supplementary Figure 3K). Overall, this line of investigation indicates that EGFR-mediated ERK signaling triggers proteasomal degradation of wild-type CIC. We also examined the ability of two *CIC* constructs carrying either the recurrent mutation affecting the HMG-box (R201W) or C1 motif (R1515H) found in oligodendrogliomas to be ubiquitylated. Interestingly, ubiquitylation of CIC was not observed in the context of these two disease-causing mutant constructs (Fig. 3m**)**. These two disease-causing mutant constructs fail to bind to the ETV5 promoter (shown by chromatin immunoprecipitation and a pull-down assay using biotinylated DNA encoding a portion of the ETV5 promoter containing the octameric motif to which CIC binds, Fig. 3n, o), yet retained their nuclear localization (Fig. 3p), suggesting that CIC’s ability to bind to the DNA is a prerequisite for ubiquitylation. In line with these findings using DNA pull-down assays we show that the ETV5 octameric oligonucleotide is robustly ubiquitylated only in cells co-transfected with wild-type CIC but neither of the two mutants (Fig. 3q). To further characterize the role of ERK and DNA binding in the degradation of CIC, we found that overexpression of ERK resulted in the release of CIC from the ETV5 DNA octamer, in a manner that was rescued by pre-treatment with PD98509 or selumetinib (AZD6244, a highly selective MEK1/2 inhibitor)^28^ (Supplementary Figure 3L-P). Corroborating this finding, ERK overexpression resulted in loss of nuclear expression of CIC in HEK293 co-transfected cells (Fig. 3r), which was abolished by pre-treatment with either PD98509 or proteasomal inhibitor MG132 (Supplementary Figure 3Q), suggesting that ERK stimulates the release of CIC from DNA to initiate ubiquitylation and subsequent degradation of CIC within the nucleus. Consistently, we found that a nuclear export inhibitor (leptomycin) substantially reduced endogenous CIC levels in NHA cells following EGF treatment (Supplementary Figure 3R), providing further support of this hypothesis. To further substantiate that DNA binding is a prerequisite for the proteasomal degradation of CIC we fused a known SRY DNA binding domain (DBD) construct to CIC wild type or CIC(R1515H) mutant resulting in DNA binding (Supplementary Figure 3S) and robust ubiquitylation of both the wild type and mutant CIC (Supplementary Figure 3S). Together these findings confirm the importance of CIC binding to its target DNA for subsequent ERK-mediated ubiquitylation.

### Proteasomal and short-term ERK inhibition rescue CIC level

**Fig. 4:**
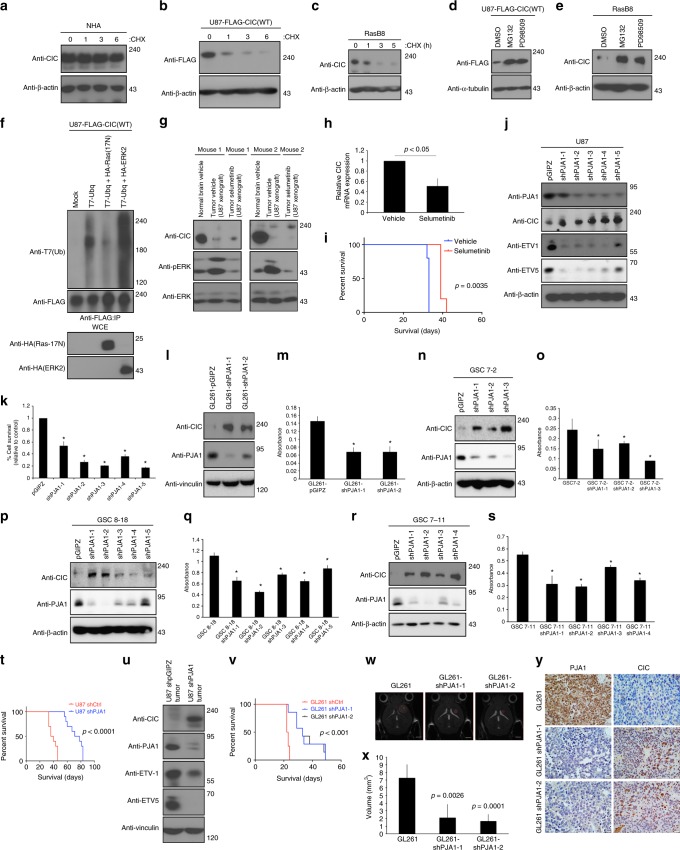
Effects of ERK inhibition and PJA1 in vitro and in vivo. **a** NHA **b** U87-Flag-CIC or **c** RasB8 treated with or without cycloheximide (CHX) lysed and immunoblotted with indicated antibodies. **d** U87-Flag-CIC or **e** RasB8 treated with or without PD98509 (1 h) or MG132 (4 h) lysed and immunoblotted with indicated antibodies. **f** U87-Flag-CIC transfected cells pre-treated with MG132, lysed and IP with anti-Flag antibody. Tumors of vehicle or selumetinib treated U87 xenograft mice lysed and **g** immunoblotted with antibodies or **h** real-time PCR analysis assessing CIC expression. **i** Kaplan–Meier survival curves of vehicle or selumetinib treated U87 xenograft mice. Log-rank statistics. Eight-week-old mice, *n* = 7 per group. U87-shPJA1-(1-5) were **j** lysed and immunoblotted with antibodies or **k** trypan blue exclusion assay conducted. GL261-shPJA1-1/2 or control **l** lysed and immunoblotted with antibodies or **m** alamar blue assay conducted. GSC 7-2-shPJA1-(1-4) or control (**n**) lysed and immunoblotted with antibodies or **o** alamar blue assay conducted. GSC 8-18-shPJA1-(1-5) (**p**) lysed and immunoblotted with antibodies or **q** alamar blue assay conducted. GSC 7-11-shPJA1-(1-4) (**r**) lysed and immunoblotted with indicated antibodies or **s** alamar blue assay conducted. **t** Kaplan–Meier survival curves of U87-shPJA1-2 or control xenograft mice. Log-rank statistics was performed. Eight-week-old mice, *n* = 10 per group. **u** Protein lysates of tumors from U87-shPJA1-2 or control xenograft mice. **v** Kaplan–Meier survival curves of GL261-shPJA1-1 or -1-2 xenograft mice. Log rank (Mantel–Cox) test, *P* < 0.001. Eight-week-old mice, *n* = 7 per group. T2 weighted anatomy MRI (**w**) imaged at 14 days post intracranial injections of GL261-shPJA1-1, -2 or controls (scale bar, 2 mm) or **x** quantified Student’s *t*-test (*P* = 0.0026, GL261 versus GL261-shPJA1-1) and (*P* = 0.0001, GL261 versus GL261-shPJA1-2). Eight-week-old mice, *n* = 7 per group. **y** Immunohistochemistry images using anti-PJA1 or -CIC antibody of tumors from GL261-shPJA1-1, -2 or control intracranial xenografts. Scale bar, 20 μm. IP Immunoprecipitated, WCE whole-cell extract. All graphs represent mean ± s.e.m. of three independent experiments performed either in octuplet for viability assays or in triplicate for real-time analysis. *P < 0.05 Student’s *t*-test compared with control. The immunoblot data are representative of at least three separate experiments

In NHA and HEK293 control cells, the half-life of CIC was >6 h (Fig. 4a and Supplementary Figure 4A). In contrast, we found CIC protein to be unstable in cultured RasB8 astrocytes, as well as in U87 cells expressing a FLAG-CIC construct (Fig. 4b, c). Proteasomal inhibition by MG132 stabilized endogenous CIC, as did short-term (1 h) ERK inhibition by PD98509 in GBM cells (Supplementary Figure 4B-D), GSCs (Supplementary Figure 4E and F), and in RasB8 astrocytes (Fig. 4e). Likewise, a dominant negative Ras mutant (Ras17N) prevented accumulation of ubiquitylated CIC in U87 cells (Fig. 4f). Although short-term ERK inhibition successfully inhibited degradation of CIC, longer-term (24 h) ERK inhibition with PD98509 or with selumetinib unexpectedly did not. We found decreases in CIC mRNA (and protein) after long-term inhibition in GBM cells and GSCs (Supplementary Figure 4G-K), suggesting that ERK activation also triggers *CIC* expression. Consistent with our in vitro findings, we treated U87 intracranial xenografts with selumetinib, which resulted in reduced *CIC* mRNA expression and protein level compared to tumors obtained from vehicle-treated mice (Fig. 4g, h). Together, these findings support the hypothesis that stabilization of CIC in combination with ERK inhibition may result in increased therapeutic effect of selumetinib in xenograft mice and increase their survival (Fig. 4i).

### PJA1 knockdown stabilizes CIC and reduces GBM proliferation

Targeting of proteins for proteasomal degradation occurs via specific E3-ubiquitin ligases of which over 600 are known in the human genome^29^. To identify the specific E3 ligase responsible for the proteasomal degradation of CIC in response to ERK activation we examined putative interactors using mass spectrometry performed on MG132-treated HA-immunoprecipitated material from HA-CIC transfected U87 cells (Supplementary Figure 4L), revealing the E3 ligase PRAJA1 (PJA1) as a possible CIC binding partner. To explore the role of this E3 ligase in CIC degradation, we conducted a knockdown of PJA1 as well as a closely related family member PRAJA2 (PJA2) in U87 and U87-EGFRvIII cells. In addition, we examined β-TrCP-1, a well-characterized E3 ligase that targets proteins with serine/threonine-phosphorylated residues^30^. Among these three candidates, only PJA1 knockdown led to stabilization of CIC protein (Fig. 4j and Supplementary Figure 4M-N). Notably, PJA1 knockdown in U87, GL261, and in three different GSCs led to stabilization of CIC protein, reduction in ETVs expression and decrease in proliferation compared to control cells (Fig. 4j–s and Supplementary Figure 4O-R). Extending these results in two different in vivo models, we found that survival of U87-shPJA1 and GL261-shPJA1-1 or GL261-shPJA1-2 xenograft mice was significantly extended compared to their respective control mice (Fig. 4t, v), which correlated with reduced PJA1 and high CIC protein levels (Fig. 4u, y and Supplementary Figure 4S). Furthermore, using T2 weighted MRI imaging we show that GL261-shPJA1-1 or GL261-shPJA1-2 bearing xenograft mice demonstrated a significant reduction in overall tumor size 14 days after injection when compared with control mice (Fig. 4w, x). Together these findings demonstrate the importance of PJA1 on tumor growth and CIC protein stability.

### ERK drives PJA1-mediated CIC degradation in GBM

**Fig. 5:**
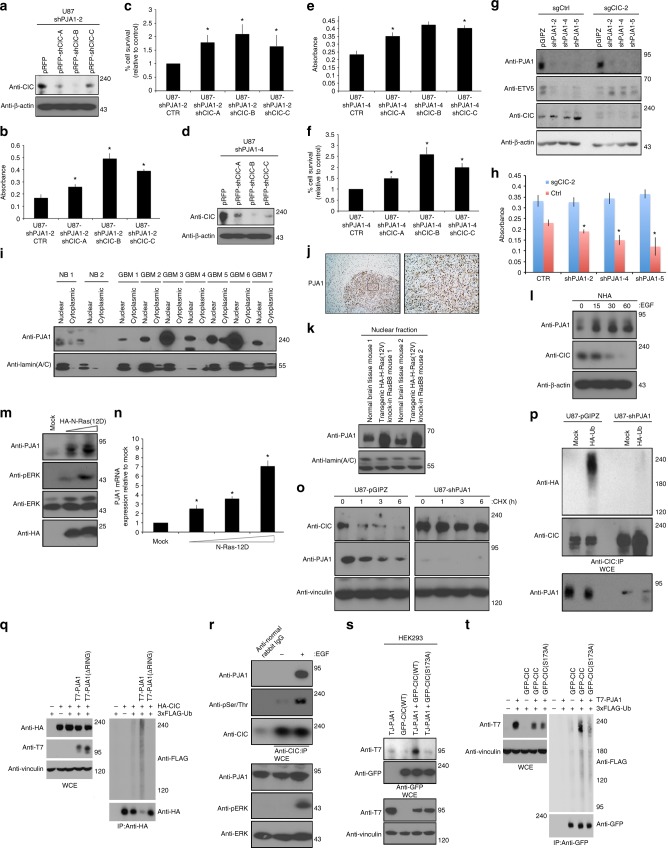
PJA1 mediates proteasomal degradation of CIC. U87-shPJA1-2-pRFP-shCIC-A/B/C were (**a**) lysed and immunoblotted with indicated antibodies or **b** alamar blue assay or **c** trypan blue exclusion assay conducted. U87-shPJA1-4-pRFP-shCIC-A/B/C was **d** lysed and immunoblotted with indicated antibodies or **e** alamar blue or **f** trypan blue exclusion assay conducted. *CIC*-null HEK93 cells, single guide (sgCIC-2) or control cells infected with shPJA1-2, -4 or -5 clones were lysed and **g** immunoblotted with indicated antibodies or **h** alamar blue exclusion assay conducted. **i** Nuclear or cytoplasmic lysates from human GBM or normal brains (NB) were immunoblotted. **j** Immunohistochemistry using anti-PJA1 antibody of U87 intracranial xenograft brains, low (scale bar, 200 μmDispase, DNAse and Pap) or high (scale bar, 50 μm) power images. **k** Nuclear lysates of high-grade RasB8 transgenic or wild-type mice immunoblotted with antibodies. **l** NHA treated with or without EGF lysed and immunoblotted with indicated antibodies. **m** HEK293 cells transfected with indicated plasmids were **n** lysed and immunoblotted with indicated antibodies or **o** real-time PCR analysis of PJA1 expression was done. **p** U87-shPJA1-2 or control cells treated with or without 100 μg/ml cycloheximide (CHX) lysed and immunoblotted with indicated antibodies. **q** U87-shPJA1-2 or control cells transfected with indicated plasmids, pre-treated with MG132 prior to 30 min EGF treatment were lysed and IP with anti-CIC antibody. **r** HEK293 cells transfected with indicated plasmids pre-treated with MG132 prior to 30 min EGF treatment were lysed and a denaturing IP using anti-HA antibody was performed. **s** HEK293 cells transfected with indicated plasmids were lysed and IP using anti-GFP antibody followed by immunoblotting with indicated antibodies. **t** HEK293 cells transfected with indicated plasmids pre-treated with MG132 or 30 min EGF treatment were lysed and a immunoprecipitation (IP) using anti-GFP antibody. UB Ubiquitin, WCE whole-cell extract, IP immunoprecipitated. Graphs represent mean ± s.e.m. of three independent experiments performed either in octuplet for viability assays or in triplicate for real-time analysis. *P < 0.05 Student’s *t*-test versus control. The immunoblot data are representative of at least three separate experiments

To further establish a role for the PJA1–CIC pathway in GBM, *CIC* knockdown in two different U87-shPJA1 clones (Fig. 5a–f) reversed the decrease in proliferation, suggesting that a shPJA1-associated decrease in proliferation was mediated by CIC stabilization. On the other hand, PJA1 knockdown in HEK293 cells in which *CIC* was deleted using CRISPR/Cas9 failed to reduce high ETV5 expression and elevated proliferation of these cells (Fig. 5g, h). These findings provide further support that PJA1’s effect on proliferation is dependent on the presence of intact CIC protein.

To further examine whether PJA1 is responsible for CIC loss in GBM we next assessed the expression of PJA1 in human GBM tumors. Lysates derived from GBM surgical specimens revealed that PJA1 protein was high compared to normal brain (Supplementary Figure 5A), as were mRNA expression levels (Supplementary Figure 5B). Importantly, PJA1 protein localized to the nucleus (Fig. 5i). We found nuclear PJA1 expression to be high in tumors of U87 xenograft mice compared to unaffected tissue (Fig. 5j) and elevated in nuclear lysates of high-grade tumors from RasB8 transgenic mice tumors compared to brains from wild-type mice (Fig. 5k). Notably, PJA1 protein was induced following EGF treatment in NHA cells (Fig. 5l) and we found increased PJA1 protein and mRNA as a result of oncogenic Ras12D expression in HEK293 cells (Fig. 5m, n). Conversely, ERK inhibition via PD98509 or MEK inhibition via selumetinib attenuated high PJA1 mRNA expression in U87 cells (Supplementary Figure 5C). To characterize this further in human tumor expression profiling data, we examined pathways correlated with PJA1-overexpression. In glioma TCGA data (GBM and Lower Grade Glioma), GSEA showed that PJA1-overexpressing GBMs were enriched for oncogenic Ras signaling pathways (Supplementary Figure 5D), including the finding that high PJA1 expression correlated with high ETV1 expression (Supplementary Figure 5E). Overall, mean PJA1 expression levels were highest in glioma samples compared to all other TCGA tumor types for which data are available (Supplementary Figure 5F), suggestive of its biological relevance in this tumor type. However, in IDH-mutant 1p/19q co-deleted low-grade gliomas (LGG) mean PJA1 expression is not statistically different between the CIC mutant versus CIC wild-type group, which is likely due to genetic loss of CIC and lack of hyperactive Ras/ERK signaling in these tumors (Supplementary Figure 5G). Consistent with our experimental observations that elevated Ras/ERK signaling was associated with PJA1 expression, we queried TCGA data for genes that were positively correlated with PJA1 expression. After identifying the top PJA1-correlated genes for each tumor type, we subjected these gene lists using functional annotation analysis (DAVID) and found that PJA1-correlated genes were significantly enriched and recurrently annotated by the keyword ‘phosphoprotein’ in the majority of TCGA tumor types (Supplementary Figure 5H). These correlative analyses from human tumors support our experimental observations that elevated Ras/ERK signaling resulted in elevated PJA1 expression. Interestingly, we found the recently identified CIC effector MMP24, shown to drive lung cancer metastasis^12^, to be elevated in PJA1-overexpressing GBM tumors (Supplementary Figure 5I) providing further support for the existence of a functional PJA1–CIC-downstream effector axis in human tumors.

### PJA1 ubiquitylates and degrades serine phosphorylated CIC

We next examined the mechanism of CIC accumulation in PJA1 knockdown cells. We found increased stability (Fig. 5o) and reduced ubiquitylation of CIC in these cells (Fig. 5p). On the other hand, PJA1-overexpressing cells displayed higher CIC ubiquitylation compared to controls (Fig. 5q), suggesting that PJA1 mediates ubiquitylation of CIC. Further support of this hypothesis came from our findings showing that PJA1 protein bound to wild-type CIC but not to either of the disease-causing mutants (Supplementary Figure 5J) that were not ubiquitylated (Fig. 3m). Importantly, deletion of the RING domain within PJA1, responsible for mediating its E3-ubiquitin ligase activity, eliminated degradation of CIC (Fig. 5q). To confirm an interaction in cells with endogenous CIC expression, we demonstrate that endogenous PJA1 interacted with endogenous CIC following EGF treatment in NHA cells (using anti-CIC antibody, Fig. 5r) as well as in HEK293 cells in which CIC was endogenously HA-tagged using CRISPR/Cas9 (using anti-HA-antibody, Supplementary Figure 5K). Since this interaction correlated with serine- and threonine-phosphorylated CIC (Fig. 5r and Supplementary Figure 5K), we examined whether EGF-mediated phosphorylation of CIC mediates PJA1 binding. We predicted that serine 173 (S173) residue on CIC, shown to be important in DNA interaction, and with flanking sequences conserved in *Drosophila*^31^, mediates PJA1 binding to CIC. Using a mutant version (S173A) of CIC incapable of phosphorylation at that residue, we found that loss of S173 phosphorylation resulted in reduced affinity for PJA1 and reduced ubiquitylation compared to wild-type CIC (Fig. 5s, t). Taken together, these results indicate that elevated Ras/ERK signaling in GBM promotes expression of the E3 ligase PJA1 to degrade phosphorylated CIC, and that the serine 173 residue on CIC has a role in this process.

### Degradation-resistant CIC increases ERK inhibition efficacy

**Fig. 6:**
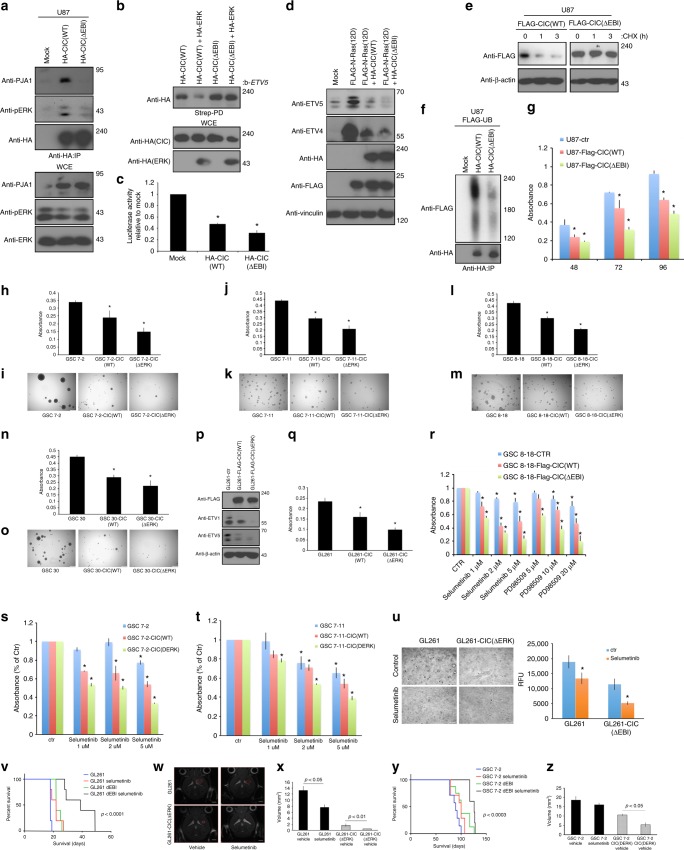
Effect of stable CIC on its function and ERK inhibition. **a** U87 transfected with indicated plasmids lysed and IP with anti-HA antibody. **b** HEK293 transfected cells lysed and Streptavidin agarose bound to biotinylated ETV5 oligonucleotide octameric motif pull-down assay was conducted. **c** Luciferase activity of HEK293 transfected cells, pGL3-ETV5 plasmid normalized to control. **d** HEK293 transfected cells lysed and immunoblotted with antibodies. **e** U87-HA-CIC or -CIC(ΔEBI) treated with or without cycloheximide lysed and immunoblotted with indicated antibodies. **f** U87 transfected cells lysed and IP with anti-HA antibody. **g** Alamar blue assay of U87-Flag-CIC, -CIC(ΔEBI) or control cells. Alamar blue assay of GSC **h** 7-2-, **j** 7-11-, **l** 8-11- or **n** 30-Flag-CIC, -CIC(ΔEBI) or controls cells. Representative images of 14 day old GSC **i** 7-2- **k** 7-11-, **m** 8-11- or **o** 30-Flag-CIC, -CIC(ΔEBI) or control cultures. Scale bar, 2 mm. GL261-Flag-CIC, -CIC(ΔEBI) or controls were **p** lysed and immunoblotted with indicated antibodies or **q** alamar blue assay conducted. **r** Alamar blue assay of GSC 8-18-Flag-CIC, -CIC(ΔEBI) or controls treated with or without DMSO, PD98509 or selumetinib. Alamar blue assay of GSC **s** 7-2- or **t** 7-11-Flag-CIC, -CIC(ΔEBI) or controls treated with or without DMSO or selumetinib. Anchorage-independent growth assay was **u** imaged (left) or quantified (right) of selumetinib treated GL261-Flag-CIC(ΔEBI). Scale bar, 200 μm. **v** Kaplan–Meier survival curves of selumetinib treated (7 days post injections) GL261-Flag-CIC(ΔEBI) or control xenograft mice. Log rank (Mantel–Cox) test, *P* < 0.0001. Eight-week-old mice, *n* = 7. T2 weighted anatomy MRI was **w** imaged 14 day post GL261-Flag-CIC(ΔEBI) injections (scale bar 2 mm) or **x** tumor volume quantified. **y** Kaplan–Meier survival curves of GSC 7-2-Flag-CIC(ΔEBI) mice treated with vehicle or selumetinib 7 days post injections. Log rank (Mantel–Cox) test, *P* < 0.0003. Eight-week-old mice, *n* = 7. **z** Quantitative T2 weighted anatomy MRI images of GSC 7-2-Flag-CIC(ΔEBI) or control mice treated with selumetinib 7 days post injections. Scale bar, 2 mm. WCE Whole-cell extract, strep-PD Streptavidin pull down, IP immunoprecipitated. Graphs represent mean ± s.e.m. of three independent experiments performed in octuplet for viability assays or in triplicate for real-time, luciferase or anchorage-independent growth assay. *P < 0.05 Student’s *t*-test versus control

Since our data suggested that CIC is continually turned over in GBM due to Ras/ERK activation, we hypothesized that deletion of the ERK-binding interface (EBI) within CIC^32^ would result in a stable CIC mutant that escapes PJA1-mediated degradation and maintains strong repressor function. We created the CIC(ΔEBI) mutant and found that this failed to interact with ERK (Fig. 6a and Supplementary Figure 6A) and retained strong repressor function even in the presence of Ras/ERK signaling (Fig. 6b–d). Importantly, we found CIC(ΔEBI) to be more stable than wild-type CIC protein and to also escape PJA1-mediated binding and ubiquitylation (Fig. 6e, f, Supplementary Figure 6B and C). Consistently, transduction of CIC(ΔEBI) significantly reduced proliferation of U87 glioma cells, RasB8 astrocytes, mouse glioma GL261 cells and GSCs (7-2, 7-11, 8-18, and 30) compared to cells expressing wild-type CIC or to mock-transduced control cells (Fig. 6g–q, Supplementary Figure 6D–H). Furthermore CIC(ΔEBI) reduced the ability of GSCs (7-2, 7-11, 8-18, and 30) to form neurospheres compared to mock-transduced control GSCs (Fig. 6i, k, m, o). Given that long-term ERK inhibition failed to stabilize CIC (Fig. 4g–i and Supplementary Figure 4G-K) we tested the effect of ERK inhibitors in the setting of stabilized CIC. We found that CIC(ΔEBI) significantly potentiated the effects of PD98509 and selumetinib on cell proliferation in cell lines stated above (Fig. 6r–u and Supplementary Figure 6I and J). Selumetinib also further reduced the ability of GL261 expressing CIC(ΔEBI) cells to grow in soft agar (Fig. 6u). Our first in vivo mouse model shows that selumetinib significantly extended median survival in U87-CIC(ΔEBI) xenograft mice compared to vehicle-treated mice (Supplementary Figure 6K). Notably, this extension in median survival with the degradation-resistant CIC mutant was double (12.5 days’ increase in survival compared to 6 days’ increase in survival) that of U87 xenograft mice treated with selumetinib (Fig. 4i). Examination of xenograft tumors showed that CIC protein was intact in U87-CIC(ΔEBI) in the setting of selumetinib treatment (Supplementary Figure 6L and M), connecting CIC stability, due to loss of its ERK-binding domain, with increased sensitivity to ERK inhibition in a mouse xenograft model. To further confirm these findings, we next employed the most frequently used syngeneic murine GBM mouse model, GL261, which is routinely used in experimental GBM therapy^26^. Selumetinib significantly extended median survival of GL261-CIC(ΔEBI) xenograft mice compared to vehicle-treated mice (Fig. 6v). This extension in median survival was almost double (39 days’ increase in survival compared to 22 days’ increase in survival) that of GL261 control xenograft mice treated with selumetinib (Fig. 6v). T2 weighted MRI imaging of GL261-CIC(ΔEBI) xenograft mice demonstrate a reduction in overall tumor size compared to GL261 control mice and show that selumetinib treatment had the greatest effect on tumor volume in the degradation-resistant CIC mutant xenograft mice compared to GL261 control mice, correlating with high CIC protein expression (Supplementary Figure 6N), which was not evident in GL261 control mice treated with selumetinib (Fig. 6w, x). Consistently, patient-derived glioma stem cells (GSC 7-2) xenograft mice show that selumetinib treatment significantly extended median survival (Fig. 6y) and decreased tumor volume (Fig. 6z) in the degradation-resistant CIC mutant xenograft mice, but not in the GSC 7-2 control mice. Together these findings demonstrate that ERK pathway inhibitors are most effective in the presence of intact CIC protein.

## Discussion

The aggressive nature of GBM has been attributed, in part, to hyperactivation of RTK and their downstream pathways, especially Ras/ERK signaling^33^, a key regulator of growth and survival^34,35^. There is need to effectively target this pathway as a strategy for therapeutic intervention^36,37^. While advances in the development of pharmacological agents directed against membrane bound RTKs and cytoplasmic kinases within the Ras/ERK pathway have been made^37,38^, results from clinical trials targeting enzymes within this pathway have been successful only in specific tumor types^4^ and to address this problem, we examined *CIC* as a potential tumor suppressor in the context of GBM, a tumor showing nearly invariable activation of the RTK/Ras/ERK pathway^39^.

We find that CIC protein levels are low/absent in GBM despite robust mRNA levels. We show that under physiological conditions, EGF stimulation triggers the rapid degradation of CIC, thereby relieving repression of its downstream targets. CIC protein is subsequently restored to basal levels to once again repress Ras/ERK target genes in the absence of signaling. We characterize this crucial regulatory mechanism and demonstrate in GBM that continuous Ras/ERK-mediated PJA1-dependent degradation and turnover of CIC results in de-repression of downstream oncogenes, including ETV gene family members. Importantly, we show that despite elevated Ras/ERK signaling, an ERK-insensitive CIC mutant (ΔEBI) maintains strong repressor function and sensitizes GBM to the effects of selumetinib (AZD6244) a highly selective MEK1/2 inhibitor^28,40^, which is currently in Phase II and III clinical trials for solid tumors, including pediatric gliomas^41^. Our findings suggest that stabilization of CIC may confer sensitivity and increase the efficacy of selumetinib in other Ras-driven cancers, including pancreatic cancer and lung cancer^12,13^.

Therapeutic efficacy from ERK inhibition would be expected to rely on intact negative regulators downstream components of this cascade. We demonstrate that in GBM, this is not the case. Long-term treatment with ERK pathway inhibitors PD98509 and selumetinib reduced *CIC* mRNA expression, providing an explanation as a contributing factor to resistance seen in these drugs for GBM. Our data suggests that ERK regulates CIC expression in two ways. Following activation, a post-translational mechanism leads to rapid degradation of CIC while a slower transcriptional mechanism leads to the production of de novo synthesis of CIC. For this reason, long-term ERK inhibition would reduce *CIC* mRNA expression, and reduce efficacy of ERK inhibitors. Interestingly, gefitinib (an EGFR inhibitor) treatment was correlated with low CIC protein expression in a non-small cell lung cancer model^11^, which may be due inhibition of *CIC* mRNA production as shown here. Thus, expression of intact CIC protein is an important requirement for RTK/Ras/ERK pathway inhibitors to be efficacious. Inhibition of elevated PJA1-mediated CIC degradation is key mechanism that could be targeted in conjunction with ERK inhibitors as a therapeutic strategy in GBM.

Many transcription factors, particularly those involved in the control of cell growth, are unstable proteins targeted for degradation by the ubiquitin-proteasome system^42^, and we find that this pathway has a key role in regulating an important tumor suppressor in cancer. More specifically, the Ras/ERK proliferative pathway promotes rapid ubiquitin-mediated degradation of nuclear CIC to allow expansion of genes involved in proliferation, and following signal completion CIC levels are restored to basal values leading to inhibition of genes involved in proliferation. An inverse relationship exists between CIC expression and cell proliferation/tumor growth, and this mechanism is analogous to other transcription factors involved in proliferation such as c-Myc that in the absence of Ras/ERK signaling is degraded via the proteasome. Normal cells regulate the cell cycle by fine-tuning the expression of both oncogenes and tumor suppressors via the ubiquitin-proteasome system. In tumors such as GBM this balance is deregulated due to hyperactive Ras/ERK signaling, favoring degradation of CIC and expression of genes such as ETV1, ETV4 and ETV5. Consistent with this hypothesis and a connection between Ras/ERK signaling and CIC function in GBM, we found that lower-grade astrocytomas, in which aberrant Ras/ERK signaling is less prominent^43^, generally display intact CIC protein expression (Fig. 1d).

It is well-established that activated ERK enters the nucleus where it phosphorylates and modulates the activity of a variety of transcription factors^44,45^, most of which are transcriptional activators involved in cell proliferation such as c-Myc^46^. We show for the first time that ERK also phosphorylates CIC to trigger its degradation by the nuclear E3 ligase PJA1. In particular, we show that serine 173 on CIC, shown previously to be involved in DNA binding^31^, is also important for PJA1 recognition and degradation. In addition to mediating phosphorylation-dependent recognition of CIC to its E3 ligase PJA1, we also show that Ras/ERK activation increases PJA1 mRNA expression. The mechanism by which this occurs is currently unknown however activation of p38α kinase, another MAPK family member, has recently been shown to induce transcriptional induction of PJA1 as well as mediating phosphorylation-dependent recognition of its target EZH2^47^. Thus, increased PJA1 mRNA expression we observe in response to Ras/ERK activation may be due to ERK-mediated transcriptional activation of PJA1.

Interestingly, the two mutant CIC constructs, found in OD, which affect the HMG-box (R201W) or C1 (R1515H) domains, fail to bind to octameric motif containing ETV5 promoter DNA and escape PJA1-mediated degradation. However, fusion of another, well known DNA binding domain of SRY, a DNA binding protein, to the CIC (R1515H) construct results in DNA binding and robust ubiquitylation. These findings demonstrate that the binding of CIC to its DNA target is a prerequisite for degradation.

It is important to note that degradation of CIC protein is not the only mechanism by which the activity of CIC is likely to be repressed in GBM. ERK-induced phosphorylation of CIC on various serine/threonine residues has been shown to promote changes in CIC localization, DNA binding ability and CIC-protein interactions with other co-repressors, primarily described in *Drosophila*^5^. Thus, the presence of an intact *CIC* gene in other tumor types does not necessarily mean that the CIC protein is a functional repressor. Loss of CIC function may be due to enhanced degradation, as shown here for GBM, or due to other phosphorylation-mediated events mentioned above, which can alter its repressor function. Identification of these key phosphorylation events on human CIC may serve as a screening tool for CIC function in tumors with intact CIC protein. Further work will examine the involvement of other effector pathways downstream of EGFR that have not been explored in the context of CIC. For example, PI3K/AKT signaling is not involved in transcriptional effects of CIC on ETV family genes^31^ but it may be involved in other recently identified CIC target genes^48^.

We uncover a role for PJA1 in GBM mediated by elevated Ras/ERK signaling, and show that PJA1 is an important downstream effector of the Ras/ERK signaling and suggest the potential importance of PJA1 in GBM that may go beyond its effect on CIC. For example, recently PJA1 has been shown to promote degradation of Enhancer of zeste homologue 2 (EZH2) in differentiating muscle cells following activation of another MAPK family member p38α kinase^47^ and EZH2 depletion has recently been shown to contribute to GBM^49^. Knockdown of CIC in U87-shPJA1 cells reversed the reduced proliferation caused by PJA1 knockdown, consistent with a role for a PJA1–CIC axis in GBM. PJA1 expression at the mRNA level was highest in glioma compared to the spectrum of TCGA samples (Figure S5F), which is consistent with recently published findings showing PJA1-overexpression in GBM^50^, further highlighting the importance of PJA1 in GBM and likely in other Ras-driven tumors and providing an opportunity for therapeutic exploration in this aggressive tumor.

**Fig. 7:**
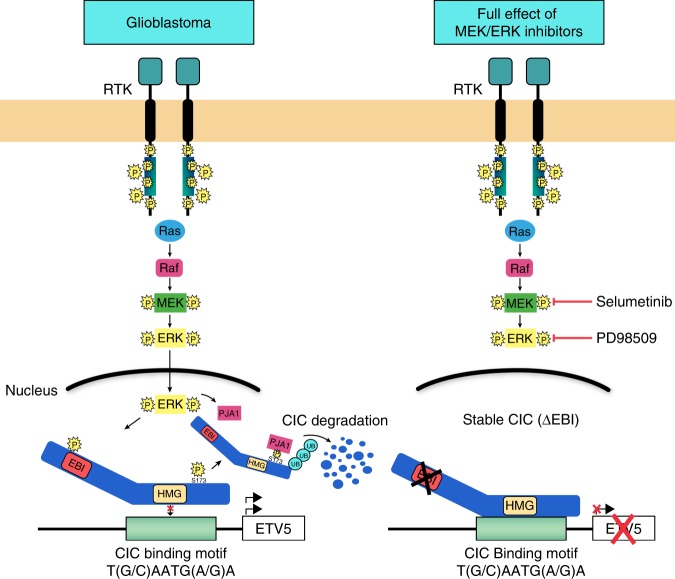
A model depicting CIC regulation in GBM and effect of the loss of CIC’s ERK-binding domain on sensitivity to ERK inhibitors

Loss of CIC facilitates activity of the Ras/ERK pathway via relief of CIC’s transcriptional repression and resultant expression of downstream targets. These data place CIC’s loss of function in the context of aberrant signaling in GBM. While *CIC* mRNA is robustly expressed in GBM, CIC protein is absent or reduced as a consequence of hyperactive Ras/ERK signaling, which triggers the activation of the nuclear E3-ubiquitin ligase PJA1 to ubiquitylate and degrade CIC and in turn promote tumorigenesis (Fig. 7). Importantly, we show that deletion of the ERK-binding interface within CIC stabilizes CIC and re-activates its tumor suppressor function in GBM, despite active upstream Ras/ERK signaling, and sensitizes GBM to the effect of selumetinib (Fig. 7). Mechanisms for CIC’s inactivation as a function of excessive Ras/ERK signaling that we characterize may be applicable to multiple cancer types. From a translational perspective, we hope our findings may stimulate further work to examine the role of CIC protein in relation to the efficacy of routinely used kinase inhibitors in GBM and other Ras-driven tumors.

## Methods

### Cells

HEK293A, HEK293T, U87, U251, U373, U118, A172, T98G, and GL261 were obtained from American Type Culture Collection. NHA, U87-vIII cell line, an EGFRvIII expression derivative of U87, RasB8 cells were previously described^27^. Cells were maintained in DMEM (Invitrogen) supplemented with 10% heat-inactivated fetal bovine serum (Wisent) at 37 °C in a humidified 5% CO_2_ atmosphere. Six GSC cultures (GSC 8-18, GSC 7-2, GSC 7-11, GSC 28, GSC 267, and GSC 30) were derived from freshly operated tumor samples from GBM patients at the University of Texas MD Anderson Cancer Center as per guidelines set by institutional review board guidelines. Each patient provided written informed consent for tumor tissues and this study was conducted under protocol LAB03-0687, which was approved by the institutional review board of the University of Texas M. D. Anderson Cancer Center^51^. GSCs and mNSC-RosaCreERT2-CIC(−/−)-41 and -42 cells were maintained as neurospheres in either defined DMEM/F12 media or neurobasal media (Gibco), respectively in the presence of growth factors EGF (20 ng/ml), recombinant basic fibroblast growth factor (bFGF) (20 ng/ml; R&D systems) and B27 growth supplement with vitamin A (1:50 working concentration; Life Technologies). To generate endogenously HA-tagged CIC in HEK293 cells the following DNA constructs were transfected: pRNAT-H1.3(Hygro), pX459-CICend and double stranded donor DNA, 5’-CCCCAGCCCTCCCCCCCACCCCCAGGTCCCTCCACAGCTGCCACAGGCAGGTACCCCTACGACGTGCCCGACTACGCCTGAGGGACCCCTGAGAAGATGCCAGGACTTATAGTACCCCCTCAGGACATGG. Cells were selected with hygromyocin and monoclonal lines were screened. To generate HEK293 CIC null cells pRNAT-H1.3(Hygro) was co-transfected with either pX330, pX459-CICsg1, or pX330-CICsg2. Cells were selected with hygromyocin and monoclonal lines were screened. To generate CIC knockdowns in NHA cells, viral supernatant was created using psPAX2, pMDG1.vsvg (gifts from Linda Z. Penn) and the following lentiviral transfer plasmids: pGIPZ (RHS4346), pGIPZ-shCIC-1 (V3LHS_358903), pGIPZ-shCIC-2 (V3LHS_358902), and pGIPZ-shCIC-3 (V2LHS_87889). Cells were selected in puromycin (Sigma) and polyclonal lines were derived. To same protocol was followed to generate PJA1, PJA2 and βTrCP knockdowns in U87 and/or U87-EGFRvIII: pGIPZ-shPJA1-1 (V3LHS_357851), pGIPZ-shPJA1-2 (V3LHS_357853), pGIPZ-shPJA1-3 (V3LHS_357850), pGIPZ-shPJA1-4 (V3LHS_357849), pGIPZ-shPJA1-5 (V3LHS_357852), pGIPZ-shPJA2-1 (V2LHS_95535), pGIPZ-shPJA2-2 (V2LHS_95534), pGIPZ-shβ-TrCP-1 (V2LHS_277057), and pGIPZ-shβ-TrCP-1-2 (V2LHS_177228). To knockdown CIC in U87-pGIPZ-shPJA1-2 or pGIPZ-shPJA1-4 cells the following transfer vectors were used to generate viral supernatant: p-RFP-CB-shLenti control (TR30033), shCIC-A (HT143147A), shCIC-B (HT143147B), shCIC-C (HT143147C). The polyclonal cells were created through selection with blasticidin (Wisent). To generate U87 and RasB8 cells that express control, FLAG-CIC(WT) or FLAG-CIC(ΔEBI) the following pMXs-GW-FLAG-IRES-BsdR transfer plasmids, along with pUMVC (Addgene 8449) and pCMV-VSV-G (Addgene 8454) were used to generate retroviral supernatants. Cells were selected in blasticidin. NSCs were isolated and cultivated as previously described with slight modifications^52^. Briefly, 1-day old CIC flox x RosaCreERT2 (BL6/N-Cictm1a(KOMP)Wtsi Gt(ROSA)26Sortm1(cre/ERT2)Tyj/Avd) mice were sacrificed and the whole brain was dissected out. The tissue was digested using Dispase, DNAse, and Papain, washed and cultured in Neurobasal Medium (Invitrogen) supplemented with 2% B27 (Invitrogen), 2 mM Glutamax (Invitrogen) and the growth factors 20 ng/ml EGF (mouse recombinant; PeproTech) and 20 ng/ml bFGF (mouse recombinant; PeproTech). After 7 days in culture neurospheres were harvested by centrifugation, dissociated mechanically, and replated. Cells were passaged once to twice weekly. To knockout CIC in mNSC, 1 µM of (Z)-4-Hydroxytamoxifen was added twice. All cell lines were routinely tested for mycoplasma infection using the PlasmoTest Kit (InvivoGen). Cell lines were not specifically authenticated.

### Plasmids

CIC cDNA was a kind gift from Paul Scotting (University of Nottingham). The cDNA was prepared for Gateway® system using a two-step PCR with primary gene specific primers (5′- CAAAAAAGCAGGCTCCACCATGTATTCGGCCCACAGGCCC-3′; 5′-CAAGAAAGCTGGGTTTCACCTGCCTGTGGCAGCTGTG-3′) and secondary AttB specific primers (5′- GGGGACAAGTTTGTACAAAAAAGCAGGCTCCACC- 3′; 5′-GGGGACCACTTTGTACAAGAAAGCTGGGTT-3′). Mutations were introduced using site directed mutagenesis with KOD polymerase (Novagen^TM^, Merck). Primers used: R201W (5′-ATGGGCCGCCAGATGTGGTCCTTCTCCCGCTT-3′; 5′-AAGCGGCACCAGGCCCTGGTCCACCAGC-3′) and R1515H (5′- CGTGAGGTGCACCAGAAGATCATGCAGGCTGC- 3′; 5′-GATCTTCTGGTGCACCTCACGGATCTTCAACTG-3’). All variants were sequence verified, using standard Gateway® sequencing primers and gene internal primers (5′-TGCCCTACCCAAGGAACGG-3′, 5′-CAGGCGCTACAGGAACTGACG-3′; 5′-CGCCTGCTTCCTCCTCAGC-3′; 5′-CCACACTTGGTGGCTGGACC-3′; 5′-TCAGTTTCTCCCGTGCAGGC-3′; 5′-GCACCCACCTCCTCAGCACC-3′; 5′-CAGAGACCTGGACTCCCACGG-3′; 5′-CCCACCCTGCAGTCTCTGGC-3′). Sequence was compared to accession number NM_015125.

Subsequently the CIC cDNA was subcloned into modified pMXs Vektors (FLAG-tag, Gateway®-cassette, IRES, BsdR) using the LR-reaction following the manufacturers protocol (Invitrogen).

To generate pX459-CICend, the following oligos were annealed and ligated into the parental vector: 5′-CACCGATAAGTCCTGGCATCTTCTC and 5′-AAACGAGAAGATGCCAGGACTTATC.

To generate pX459-CICsg1, the following oligos were annealed and ligated into the parental vector: 5′-CACCGCCCCTCCGTGCAGCCGAGCG and 5′AAACCGCTCGGCTGCACGGAGGGGC. To generate pX330-CICsg2, the following oligos were annealed and ligated into the parental vector: 5′-CACCGCGACGTTTTCCGGGCGGTAG and 5′-AAACCTACCGCCCGGAAAACGTCGC. pCMV5-HA-CIC (DU19108), pcDNA5-FRT/TO-GFP-CIC (DU16689) and pcDNA5-FRT/TO-GFP-CIC(S173A) (DU16902) were purchased from MRC-PPU University of Dundee. HA-N-Ras(17N and 12D) and FLAG-N-Ras(G12D) were generated earlier^53^. HA-ERK was purchased from Addgene (8974). FLAG-PJA1 plasmid was obtained from Dr. Mikko Taipale.

### Xenograft models of GBM

All animal procedures were carried out according to animal user protocols approved ethically by the Institutional Animal Care Committee under the guidelines of the Canadian Council on Animal Care and the University Health Network Research Ethics Board. C57/BL6 (for GL261 implantation) or NOD/SCID (male 8 weeks) were anaesthetized using 0.5 mg/g of intraperitoneal injection of 20 mg/ml Avertin (Sigma-Aldrich) and 5 mg/kg of the pre-surgical analgesic Anafen 1 mg/ml (Ketoprofen) was administered subcutaneously. TearGel(Novartis) was applied to the eyes to prevent corneal dehydration and abrasion. Once a toe pinch no longer elicited a response, the scalp was cleaned and hair removed, and a midline incision was made from the ears to the eyes. Underlying periosteum was frozen with 2% Lidocaine (Bimeda MTC) and removed with scissors. Mice were placed on a digital stereotaxic frame and from bregma the cortex coordinates were identified (X:1.6, Y:1, Z:0.6). A high-speed dental drill with a 0.7-mm adaptor (Fine Science Tools) was used to bore a whole through the skull. 1 × 10^5^ cells resuspended in 10 ml of sterile 1× PBS were injected 3 mm deep through the hole using a 10-ml 30-G Hamilton microsyringe, over a 1-min period. Mice were sutured and returned to a fresh sterile heated cage to recover and supplemented with 0.3 mg/ml Enrofloxacin in the drinking water (Baytril Bayer/CDMV, cat. no.102207). Treatment with selumetinib in vehicle (4% DMSO + 30% PEG 300 + 5% Tween 80 + ddH2O) or vehicle alone was administered 7 days post engraftment at 100 mg/kg/day via oral gavage^54,55^.

### Transgenic mouse models

GBM transgenic mouse model, RasB8, was generated through integration of a V12 RasB8 mutation under the control of GFAP-promoter leaving mice predisposed to sporadic GBM-like astrocytoma^27^. Embryonic stem cell complementation methodology was used to integrate a V12 RasB8 mutation (IRES LACz) under the control of GFAP-promoter into an ICR background strain mouse. The positive RasB8 male mice are bred heterozygously to ICR females, as the homozygous crosses are lethal before P14^27^. Genotyping is carried out for both the RasB8 mutation and the LACz reporter construct; in addition, LAC immunohistochemistry (IHC) is carried out to ensure full protein translation.

### MRI

A 7-Tesla Bruker model BioSpec 70/30 MR system with B-GA12 gradient coil, 7.2 cm diameter linear radiofrequency transmitter coil, murine head radiofrequency receiver coil and murine slider bed was used for serial imaging. Mice under isoflurane anesthesia were positioned on the MR bed with a bite block and water warming system to maintain body temperature during imaging. Serial multiparametric MRI protocol was carried out as previously described^56^.

### Patient-derived tumor samples

Patient resected samples were obtained from Toronto Western Brain Tumor Bank and processed in accordance with a University Health Network Research Ethics Board-approved protocol.

### Quantitative real-time RT-PCR

Total RNA was isolated using the Qiagen RNeasy mini kit according to the manufacturer’s recommendations. One μg RNA was reverse transcribed to cDNA using the QuantiTect Reverse Transcription Kit (Qiagen) and quantitative real-time PCR performed was performed using TaqMan universal master mix II (Life Technologies) according the manufacturers protocol with a StepOne Real-Time PCR machine (Life Technologies). Human CIC (Hs00209424_m1), PJA1 (Hs00254654_s1), ETV5 (Hs00927557_m1), ETV1 (Hs00951951_m1), and β-actin (Hs01060665_g1) and mouse ETV1 (Mm00514804_m1) were purchased from Life Technologies.

### Chromatin immunoprecipitation (ChIP)

HEK293 transfected cells were cross-linked with 1% formaldehyde at 37 °C for 10 min. Cells were rinsed with ice-cold PBS plus 5% BSA followed by PBS and harvested with PBS plus 1 × protease inhibitor cocktail (Roche Molecular Biochemicals, IN). Harvested cells were centrifuged for 2 min at 1000 × *g*. Cells were lysed in 0.35 ml of lysis buffer (1% SDS, 10 mM EDTA, 50 mM Tris–HCl, pH 8.1, 1 × protease inhibitor cocktail) by sonication (Diagenode Biorupter). The lysed cells were subjected to centrifugation at maximum speed for 15 min. Supernatants were collected and diluted in dilution buffer (1% Triton X-100, 2 mM EDTA, 150 mM NaCl, 20 mM Tris–HCl, pH 8.1). Anti-FLAG antibody (Sigma, F1804) was prebound for 6 h to protein A and protein G Pierce magnetic beads (Thermo Fisher Scientific) and washed three times with ice-cold PBS plus 5% BSA and then added to the diluted chromatin for overnight immunoprecipitation. The magnetic bead-chromatin complexes were collected and washed six times in RIPA buffer (50 mM HEPES [pH 7.6], 1 mM EDTA, 0.7% Na deoxycholate, 1% NP-40, 0.5 M LiCl) and then washed twice with TE buffer. Cross-linking was reversed with decrosslinking buffer (1% SDS, 0.1 M NaHCO3) overnight at 65 °C. DNA fragments were purified with a QIAquick Spin Kit (Qiagen, CA) followed by quantitative PCR (ChIP-qPCR) on the promoter of ETV5 using the following primers *ETV*5-F: 5′-ATA ACT TTG CTT GGT GCT GCA GCT GCG-3′ and *ETV5*-R: 5′-CCA TTG GCC AAT CAG CAC AGG CTTG-3′^57^. Fold enrichment was calculated over input.

### Luciferase assay

Cells were transfected in triplicate with pGL3-ETV5 plasmid, which contains four consensus CIC octameric motifs, and pRL-SV40 (Promega) Renilla luciferase control. Cells were transfected with the indicated plasmids and lysates were assayed for luciferase activity. A dual-luciferase reporter assay system (Promega) was used, and luminescence was measured using a GloMax 20/20 luminometer (Promega). Relative light units (RLU) from firefly luciferase were normalized against Renilla luciferase values.

### Immunoprecipitation and oligonucleotide pull-down assay

For immunoprecipitation and western blotting cells were harvested in EBC lysis buffer (50 mM Tris, pH 8, 120 mM NaCl, 0.5% NP-40) and supplemented with protease inhibitors (Roche). Lysates were immunoprecipitated using the indicated antibodies along with protein A-Sepharose (Repligen). Bound proteins were washed five times in NETN buffer (20 mM Tris, pH 8, 100 mM NaCl, 1 mM EDTA, 0.5% NP-40), eluted by boiling in sample buffer and resolved by SDS–polyacrylamide gel electrophoresis. Proteins were electro-transferred onto polyvinylidene difluoride membrane (Bio-Rad), blocked and probed with the indicated antibodies. Uncropped scans of the most important blots are presented as a supplementary figure in the Supplemental Information section. For experiments in which the nuclear or cytoplasmic fractions were prepared the nuclear extraction kit (Cayman Chemicals) was used according to the manufacturer’s instruction and immunoblotting was performed as stated above. For denaturing immunoprecipitations used to detect ubiquitylation lysates were supplemented with 1% SDS and boiled for 5 min to denature proteins and disrupt interactions. The boiled lysates were diluted so the final concentration of SDS was less than 0.1% and immunoprecipitation was performed. For oligonucleotide pull-down assay the following oligos were annealed ETV5-WT octameric repeat: 5′-Biotin-CGCGTTTTTTATGAATGAAAAACGTCCTTA and 5′-TAAGGACGTTTTTCATTCATAAAAAACGCG or for ETV5-Mutant octameric repeat the following oligos were annealed: 5′-Biotin-CGCGTTTTTTATTAAAAGGAAACGTCCTTA and 5′-TAAGGACGTTTCCTTTTAATAAAAAACGCGC. 1 μg of annealed oligonucleotides was bound to streptavidin agarose (Thermo) and mixed with lysate for 2 h. Bound proteins were resolved as indicated above. For experiments in which DNA binding domain (DBD) of SRY was fused to CIC, nucleotides encoding the DBD of SRY (cDNA encoding SRY was purchased from Origene) were PCR amplified with primers containing *Eco*RI sites on both ends. pCMV5-HA-CIC(WT) and pCMV5-HA-CIC(R1515H) were each linearized using *Eco*RI. The SRY insert was ligated into each vector. The constructs were sequenced to ensure correct directionality of SRY(DBD). DNA pulls down assays were conducted as stated above.

### Antibodies

The following antibodies were obtained from Cell Signaling Technologies: HA (C29F4) (1:6000), Lamin(A/C) (2032), pERK (4370), ubiquitin (3933), PJA2 (40180), and β-TrCP (D13F10), β-actin (8H10D10) (1:20,000), myc tag (71D10), and α-tubulin (2144) (1:5000). pERK (sc-7383 and sc-16982-R), PJA1 (sc-517068), and GFP (sc-9996) (1:6000) were obtained from Santa Cruz Biotechnology. Ki67 was obtained from Dako. Phospho Ser/Thr (ab17464), T7 tag (ab9138), capicua (ab123822), ETV1 (ab81086), Renilla (ab187338), and myc protein (ab3207) were obtained from Abcam. The following antibodies were purchased from Millipore T7 tag (69522), capicua (ABN446) and capicua (MABN449). FLAG-M2 (F1804), β-actin (A5316) (1:10,000), vinculin (V9264) (1:30,000), ETV5 (WH0002119M2), and polyclonal ERK (M5670) antibodies (1:5000) were obtained from Sigma. PJA1 (MBS153701) was purchased from MyBioSource.com. All antibodies were utilized at a 1:1,000 dilution unless otherwise specified.

### Chemicals and reagents

MG132 (IZL-3175-v) was obtained from Peptides International. PD98509 (P215), leptomycin B(L2913), (Z)-4-Hydroxytamoxifen (H7904), cycloheximide (C4859) methyl cellulose (M0262), Tween 80 (P1754) and chloroquine (C6628) were obtained from Sigma. EGF was purchased from Gibco. Streptavidin agarose resin was obtained from Thermo Scientific (20349). Selumetinib (A8207) was purchased from ApexBio.

### Cell proliferation and neurosphere assays

Equal numbers of cells were plated in triplicate in 96-well plates and cellular proliferation was assessed using Alamar Blue proliferation assay as per manufacturer’s instructions (Invitrogen) or 5-bromodeoxyuridine cell proliferation assay as per manufacturer’s instructions (BioVision). Trypan blue exclusion assay was performed by directly counting cells plated in triplicate using the Beckman Coulter Vi-CELL (12-Sample Carousel) Cell Viability Analyzer (Beckman Coulter). For drug sensitivity assays 5000 cells were seeded in 96-well plates and 24 h later the cells were treated with increasing concentrations of drugs and Alamar Blue assay was performed. Additionally, 24 h post transfection with indicated plasmids cells were labeled with Cell Proliferation Dye eFluor 670 (eBioscience) per manufacturer’s instruction. At indicated time points, dilution of the dye, and hence, proliferation was assessed by flow cytometry at the Core Flow Cytometry Facility of the Hospital for Sick Children. All the proliferation assays were repeated at least in triplicates. To assess neurosphere formation ability, GSCs were seeded at a density of 1000 cells per well in 6-well plates in triplicate and the resulting spheres were counted 10–14 days later.

### Anchorage-independent transformation assay

Anchorage-independent transformation assays were performed by using the Cyto-Select 96-Well Cell Transformation Assay Kit (Cell Biolabs). Briefly, the cells were plated in soft agar in a 96-well plate at 2000 cells per well. The culture medium was changed every two days, and the cells were in culture for 10 days. To measure anchorage-independent growth, the agar layers were dissolved and lysed. Ten microliters of the lysed solutions of each well was mixed with CyQuant, and the fluorescence was read.

### Immunohistochemistry

Paraffin-embedded blocks were cut in 5 μm sections. Slides were processed as follows: they were dewaxed in xylene followed by rehydration in a standard alcohol series. Antigen retrieval was done by pressure-cooking for 20 min in citrate buffer (pH 6.0), followed by blocking of endogenous peroxidase in 0.3% H_2_O_2_. 10% serum derived from the secondary antibody source was used to block for 30 min. Sections were incubated overnight at 4 °C with indicated primary antibodies. Antibodies were detected using a secondary HRP-labeled mouse or rabbit antibody detection system (Dako EnVision + System-HRP k4401, k4403) followed by addition 3,3′-diaminobenzidine (DAB) chromagen (Vector Labs) for visualization. Sections were counterstained with hematoxylin (Fisher Scientific Inc.) and slides were dehydrated in 70, 80, and 100% ethanol and xylene. Slides were covered by coverslips and mounted in Permount (Fisher Scientific Inc.). All images were captured on an Olympus IX73 fluorescence microscope system and analyzed using CellSens Dimension software.

### Immunofluorescence

Transfected HEK293 cells grown on coverslips in 6-well plates were fixed in cold 100% methanol at −20 °C for 30 min and blocked with 1% normal goat serum for 1 h at room temperature. The cultures were then incubated for 1 h with either 10 μg/ml of polyclonal antibody to FLAG. All of the cultures were then incubated for an additional hour with fluorescein-conjugated goat anti-mouse. The nuclei were counterstained with DAPI. Secondary antibody alone was used as a control. All of the cultures were then mounted in elvanol and examined with an Olympus IX73 fluorescence microscope system and analyzed using CellSens Dimension software.

### TCGA and microarray data analysis

Publically available level 3 gene expression data (RNAseq) from TCGA was analyzed for gene expression levels and PJA1-correlated genes. Total RNA was isolated from CIC transfected U87 and HEK293 cells and processed for microarray analysis at the Princess Margaret Genomic Centre, University Health Network. Briefly, the gene expression microarray data were measured using the Affymetrix®GeneChip® (Probe Array type: HuGene-2_0-st). We performed log2 transformation, quantile normalization and Robust Multiarray Average (RMA) background correction beside some additional quality checks using Partek GenomicsSuite software (Partek, St. Louis USA). We split the four samples into two groups of U87 and HEK293 cells, and then calculated fold changes between CIC-transfected U87 and HEK293 versus corresponding controls for U87 and HEK293.

### Statistical analysis

All experiments were performed at least in triplicate with mean and standard error of the mean (s.e.m.) reported. Log-rank statistics were performed on survival curves. For direct comparisons, an unpaired Student’s *t*-test was carried out. Significance was defined as *P < 0.05 unless specifically stated in figure legends.

### Reporting summary

Further information on experimental design is available in the Nature Research Reporting Summary linked to this article.

## Supplementary information

Supplementary Information

Reporting Summary

## Data Availability

The data supporting the findings of this study are available from the corresponding authors upon reasonable request. The microarray data can be obtained using the following at the Gene Expression Omnibus (GEO) accession code GSE123444.
