# Supplementary material for: CIC protein instability contributes to tumorigenesis in glioblastoma
Source: Nat Commun. 2019 Feb 8;10:661. doi: 10.1038/s41467-018-08087-9 (PMC6368580; doi:10.1038/s41467-018-08087-9)

Supplementary Information

CIC protein instability contributes to tumorigenesis in glioblastoma

Bunda et al., 2018

SUPPLEMENTARY FIGURES

Supplementary Figure 1

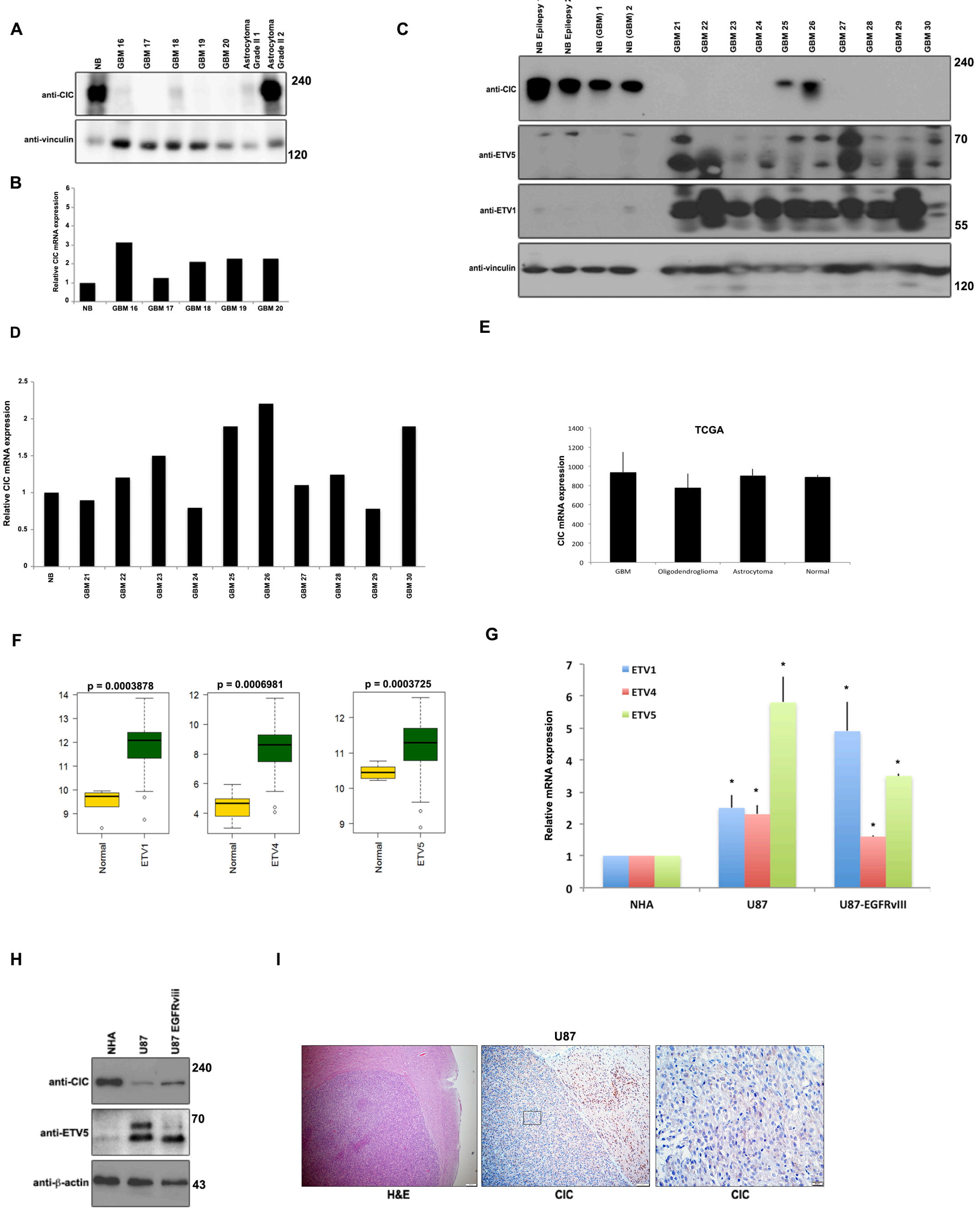

Supplementary Figure 1

Expression of CIC and its targets in human gliomas and cells. Human operative GBM samples or normal derived brains (NB) were lysed and (**A** and **C**) immunoblotted with indicated antibodies (**B** and **D**) or total RNA extracted and quantitative real-time PCR analysis was carried out using TaqMan gene expression assays. The graph depicts fold changes in CIC expression relative to normal brain. (**E**) CIC mRNA expression in GBM, oligodendroglioma, astrocytoma and normal brain obtained from the cancer genome atlas (TCGA) database. (**F**) ETV1, 4 and 5 mRNA expressions in GBM and normal brain according to TCGA. (**G**) Total RNA was extracted from NHA, U87 and U87-EGFRvIII cells and quantitative real-time PCR analysis was carried out using TaqMan gene expression assays. The graph depicts fold changes in ETV1, 4 and 5 mRNA expression relative NHA. Data represent mean ± s.e.m. of three independent experiments performed in triplicate. *P<0.05 Student's *t*-test compared to NHA for each gene. (**H**) NHA, U87 or U87-EGFRvIII cells were lysed and immunoblotted with indicated antibodies. (**I**) Representative images of hematoxylin and eosin (H&E) staining and immunohistochemistry using anti-CIC antibody of sections obtained from brains of intracranial U87 xenograft mice. Scale bar, 1 mm (left) and 50 μm (right). The immunoblot data are representative of at least three separate experiments.

Supplementary Figure 2

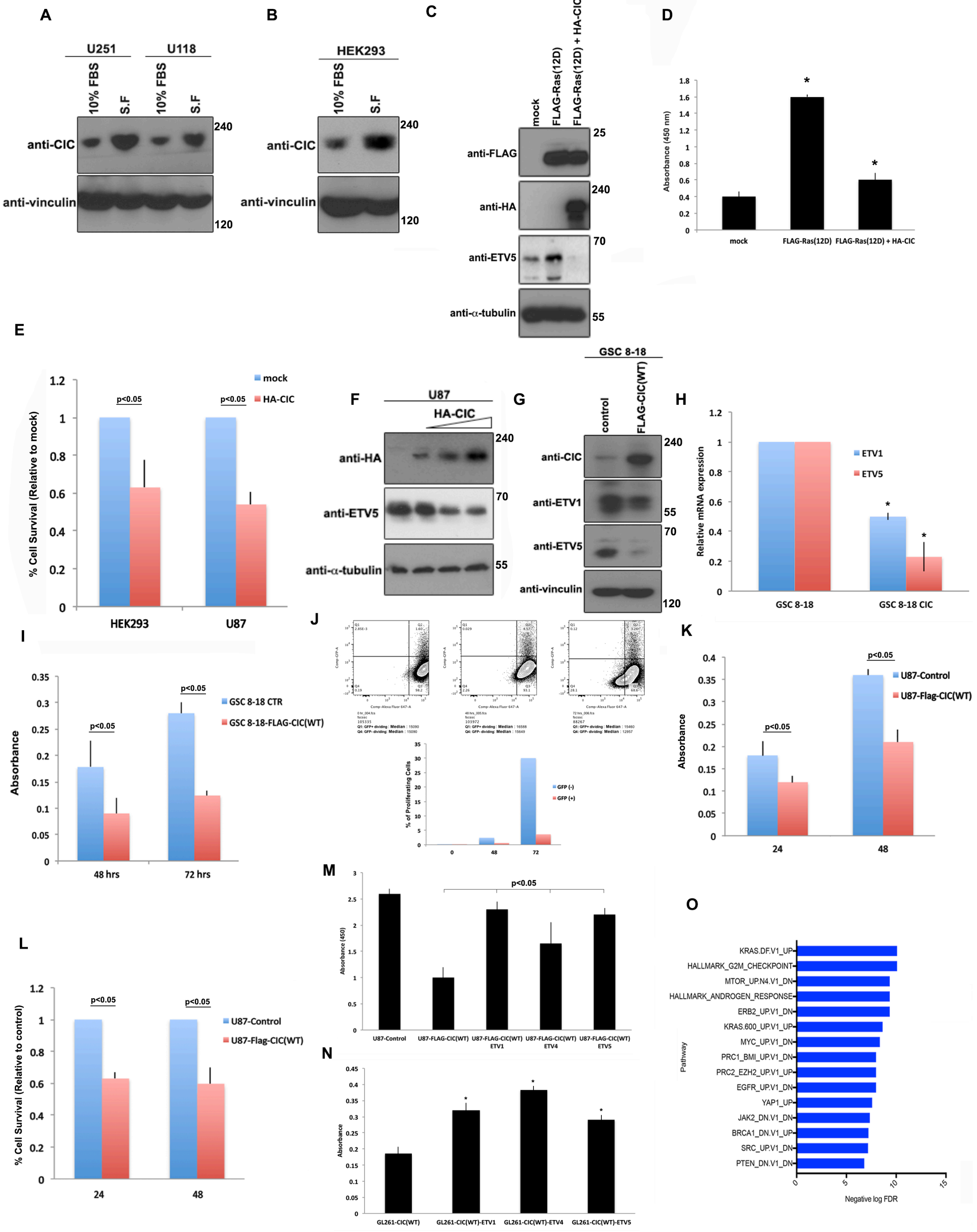

Supplementary Figure 2

Cell growth and viability as a function of CIC expression. U251, U118 (**A**) or HEK293 (**B**) cells were either serum starved or maintained in 10% fetal bovine serum (FBS) for 24 hours were lysed and immunoblotted with indicated antibodies. HEK293 cells transfected with the indicated plasmids were lysed (**C**) and immunoblotted with the indicated antibodies or (**D**) cell proliferation was assessed by BrdU incorporation assay. Data represent mean±s.e.m. of three independent experiments performed in triplicate. *$P<0.05$ Student's *t*-test compared with mock control. (**E**) Equal number of HEK293 or U87 cells transfected with HA-CIC or empty plasmid controls were plated and cell viability was assessed at indicated time points (in hours) using trypan blue exclusion assay. Data represent mean ± s.e.m. of three independent experiments performed in octuplet. (**F**) U87 cells transfected with increasing concentrations of HA-CIC or empty plasmid control were lysed and protein immunoblotted with the indicated antibodies. Equal number of glioma stem cells (GSC 8-18) stably expressing FLAG-CIC or empty plasmid controls were lysed and (**G**) protein lysates were immunoblotted with indicated antibodies or (**H**) or total RNA extracted and quantitative real-time PCR analysis was carried out using TaqMan gene expression assays. The graph depicts fold changes in ETV1 or ETV5 expression relative to controls. Data represent mean ± s.e.m. of three independent experiments performed in triplicate. *$P<0.05$ Student's *t*-test compared with control. (**I**) Equal number of glioma stem cells (GSC 8-18) stably expressing FLAG-CIC or empty plasmid controls was lysed and alamar blue proliferation assay conducted at indicated time points (in hours). Data represent mean ± s.e.m. of three independent experiments performed in octuplet. (**J**) U87's transfected with GFP-CIC labeled with the

eFluor 670 proliferation dye were analyzed by flow cytometry at indicated time points. Graphs depict percentage of proliferating GFP-CIC versus GFP-negative cells within the same experimental plate. Data are representative of at least three independent experiments performed in triplicate. Equal number of U87 stably expressing cells Flag-CIC(WT) or empty plasmid controls were plated and cell viability was assessed at indicated time points (in hours) using (**K**) alamar blue or (**L**) trypan blue exclusion assay. Data represent mean ± s.e.m. of three independent experiments performed in octuplet. Equal number of U87 stably expressing Flag-CIC(WT) (**M**) or (**N**) GL261 stably expressing Flag-CIC(WT) where either transfected with or without either ETV1, 4 or 5, lysed and cell proliferation was assessed by BrdU incorporation assay. Data represent mean ± s.e.m. of three independent experiments performed in octuplet. *P<0.05 Student's t-test compared with control. (**O**) Gene set enrichment analysis (GSEA) of concordant genes down-regulated with a minimum fold change of 1.3 in both HEK293 and U87 cells transfected with CIC showing oncogenic molecular signature pathways of which 6/15 (40%) were directly related to RTK/Ras/Raf/ERK pathways. The immunoblot data are representative of at least three separate experiments.

Supplementary Figure 3

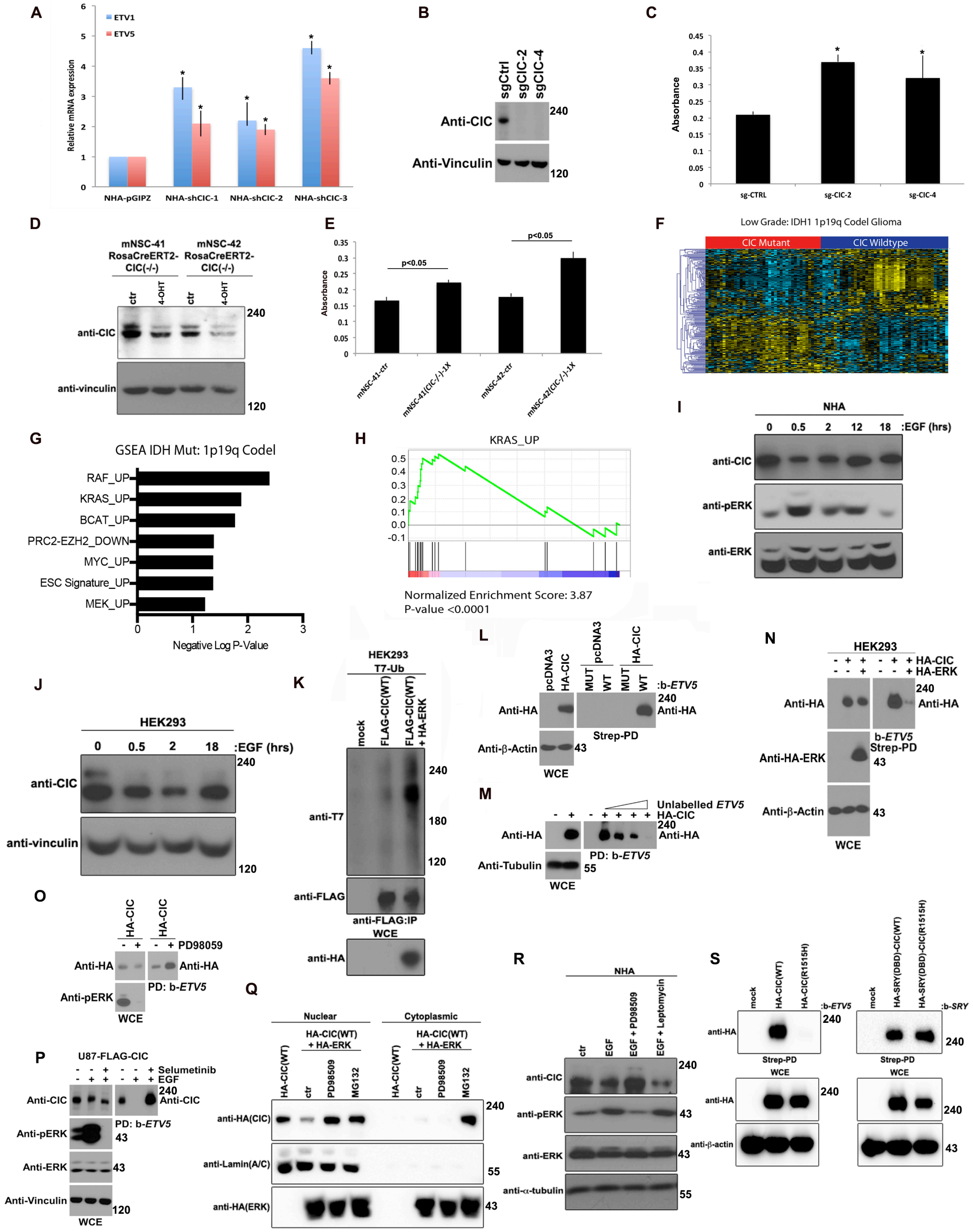

Supplementary Figure 3

CIC knockdown, stabilization, and pathway analysis in human tumors. (**A**) NHA infected with pGIPZ (control) or shCIC clones 1, 2 or 3 lentivirus were lysed and total RNA extracted and quantitative real-time PCR analysis was carried out using TaqMan gene expression assays. The graph depicts fold changes in ETV1 or ETV5 expression relative to the pGIPZ controls. Data represent mean ± s.e.m. of three independent experiments performed in triplicate. *$P<0.05$ Student's *t*-test compared with control. Equal number of two different *CIC*-null HEK93 cells generated with two different single guide (sg) RNA or HEK293 sg control (CTRL) were lysed (**B**) and immunoblotted with indicated antibodies or (**C**) were plated and cell viability was assessed using trypan blue exclusion assay. Data represent mean ± s.e.m. of three independent experiments performed in octuplet. *$P<0.05$ Student's *t*-test compared to sg-control (CTRL) control. (**D**) Two different mouse neural stem cells (mNSC) homozygous conditional for CIC in a RosaCreERT2 background (mNSC-RosaCreERT2-CIC(-/-)-41 and -/-42) treated once with or without (Z)-4-Hydroxytamoxifen (4-OHT) were lysed and immunoblotted with indicated antibodies. (**E**) Equal numbers of mNSC-RosaCreERT2-CIC(-/-)-41 or -/-42 treated once (1X) with or without (Z)-4-Hydroxytamoxifen were plated and alamar blue assay was performed. Data represent mean ± s.e.m. of three independent experiments performed in octuplet. (**F**) Gene-expression analysis of *CIC*-mutants versus wild type IDH mutant 1p and 19q co-deleted low-grade gliomas identified 221 differentially expressed genes (fold cutoff of 1.5, p-value <0.05 and FDR<10%). (**G**) Gene-set enrichment analysis of *CIC*-mutant group versus wild type IDH mutant 1p19q co-deleted gliomas (**H**) K-Ras signaling is significantly up-regulated in *CIC*-mutant versus *CIC*-wild

type 1p19q co-deleted oligodendroglioma, as well as in GBM. (**I**) NHA or (**J**) HEK293 cells treated with or without EGF for indicated time points were lysed and immunoblotted with indicated antibodies. (**K**) HEK293 cells transfected with indicated plasmids were pre-treated with MG132 lysed, immunoprecipitated (IP) with anti-FLAG antibody and immunoblotted with indicated antibodies. Whole cell extract (WCE) (**L**) HEK293 cells transfected with the indicated plasmids were lysed and an equivalent amount of lysate was incubated with Streptavidin agarose bound to biotinylated oligonucleotides with either wild type or mutant CIC octameric motif derived from ETV5 motif. Bound proteins were detected by immunoblotting. Streptavidin pull down (strep-PD); whole cell extract (WCE). (**M**) HEK293 cells transfected with the indicated plasmids were lysed and an equivalent amount of lysate was incubated with Streptavidin agarose bound to biotinylated CIC octameric motif oligonucleotides derived from ETV5 or with increasing amounts of non-biotinylated ETV5 oligonucleotide. Bound proteins were detected by immunoblotting. Streptavidin pull down (strep-PD); whole cell extract (WCE). HEK293 cells transfected with the indicated plasmids (**N**) or (**O**) treated with or without 1 hour PD98509 were lysed and an equivalent amount of lysate was incubated with Streptavidin agarose bound to biotinylated ETV5 oligonucleotide. Bound proteins were detected by immunoblotting. Streptavidin pull down (strep-PD); whole cell extract (WCE). (**P**) U87-Flag-CIC(WT) cells pre-treated for 1 hour with selumetinib in the presence or absence of EGF (30-minute treatment) were lysed and an equivalent amount of lysate was incubated with Streptavidin agarose bound to biotinylated CIC octameric motif oligonucleotides derived from ETV5 or with increasing amounts of non-biotinylated ETV5 oligonucleotide. Bound proteins were detected by immunoblotting.

Streptavidin pull down (strep-PD); whole cell extract (WCE). (**Q**) Nuclear or cytoplasmic fractions were isolated from HEK293 cells transfected with indicated plasmids, treated with or without either PD98509 or MG132 and were immunoblotted with indicated antibodies. (**R**) NHA cells pre-treated with PD98509 or leptomycin and treated with or without 30 minute EGF were lysed and immunoblotted with indicated antibodies. (**S**) HEK293 cells were transfected with indicated plasmids were lysed and an equivalent amount of lysate was incubated with Streptavidin agarose bound to either (left panel) biotinylated ETV5 oligonucleotides octameric motif or (right panel) biotinylated SRY DNA binding domain (DBD) motif, bound proteins were detected by immunoblotting. Streptavidin pull down (strep-PD); Whole cell extract (WCE). The immunoblot data are representative of at least three separate experiments.

Supplementary Figure 4

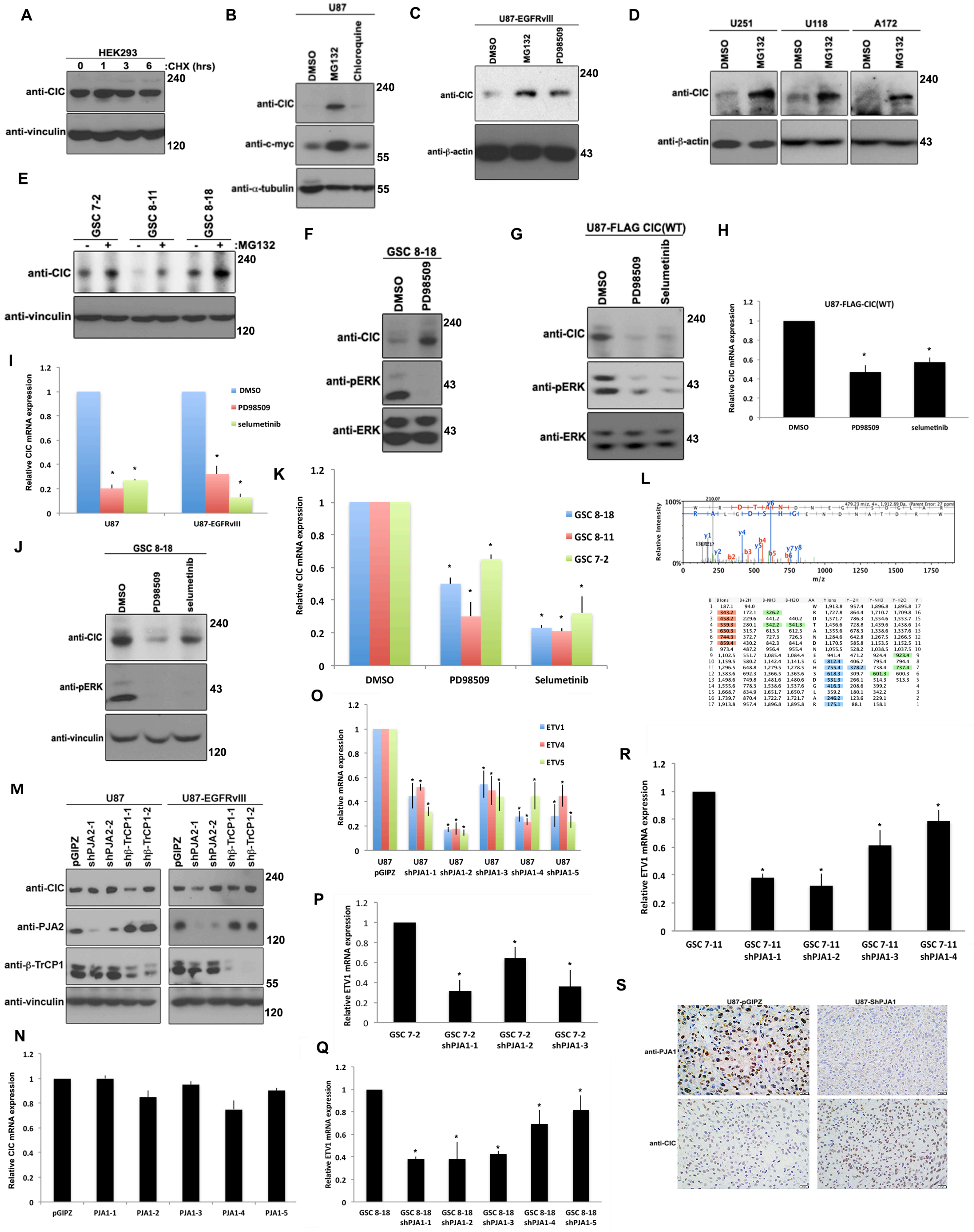

Supplementary Figure 4

CIC expression in relation to Ras/ERK activation and effects of PJA1 expression.

(**A**) HEK293 cells treated with or without cycloheximide (CHX) for indicated time points were lysed and immunoblotted with indicated antibodies. (**B**) U87 cells pre-treated with or without MG132 for 4 hours or choloroquine were lysed and immunoblotted with indicated antibodies. (**C**) U87-EGFRvIII, (**D**) U251, U118, A172 or (**E**) GSC 7-2, 8-11, 8-18 treated with or without MG132 for 4 hours were lysed and immunoblotted with indicated antibodies. (**F**) GSC 8-18 treated with or without PD98509 or DMSO control for 1 hour were lysed and immunoblotted with indicated antibodies. U87-FLAG-CIC(WT) cells treated with or without PD98509 or selumetinib for 24 hours were lysed (**G**) and immunoblotted with indicated antibodies or (**H**) total RNA extracted and quantitative real-time PCR analysis was carried out using TaqMan gene expression assays. The graph depicts fold changes in CIC expression relative to the DMSO control. Data represent mean ± s.e.m. of three independent experiments performed in triplicate. *P<0.05 Student's t-test compared with DMSO. (**I**) U87 or U87-EGFRvIII cells treated with or without DMSO, PD98509 or selumetinib for 24 hours were lysed and total RNA extracted and quantitative real-time PCR analysis was carried out using TaqMan gene expression assays. The graph depicts fold changes in CIC expression relative to the DMSO control. Data represent mean ± s.e.m. of three independent experiments performed in triplicate. *P<0.05 Student's t-test for each cell line compared with DMSO. (**J**) GSC 8-18 treated with or without DMSO, PD98509 or selumetinib for 24 hours were lysed and immunoblotted with indicated antibodies. (**K**) GSC 8-18, 8-11 or 7-2 treated with or without DMSO, PD98509 or selumetinib for 24 hours were lysed total RNA

extracted and quantitative real-time PCR analysis was carried out using TaqMan gene expression assays. The graph depicts fold changes in CIC expression relative to the DMSO control. Data represent mean ± s.e.m. of three independent experiments performed in triplicate. *P<0.05 Student's *t*-test compared with DMSO for each GSC. (**L**) Mass spectrometry analysis identifies PJA1 as a CIC binding partner. Cells were transfected with CIC-FLAG and treated with MG132 for 6 hours prior to lysis. CIC was affinity captured on FLAG beads and subjected to trypsin digestion. The resulting peptides were subjected to LC-electrospray ionization-MS/MS. Spectrum-sequence alignment of PJA1 peptide, top panel. Expected and observed fragment masses, bottom panel. (**M**) U87 or U87-EGFRvIII cells infected with pGIPZ (control), shPJA2 or sh-B-TrCP1 clones 1 and 2 lentivirus were lysed and immunoblotted with the indicated antibodies. U87s infected with pGIPZ (control) or shPJA1 clones 1-5 lentivirus were lysed and total RNA extracted and quantitative real-time PCR analysis was carried out using TaqMan gene expression assays. The graph in (**N**) depicts fold changes in CIC expression relative to the pGIPZ controls, the graph in (**O**) depicts fold changes in ETV1, 4 or 5 mRNA expression relative to pGIPZ controls. Data represent mean ± s.e.m. of three independent experiments performed in triplicate. *P<0.05 Student's *t*-test compared with control. Indicated control or shPJA1 clones in GSC 7-2 (**P**) GSC 8-18 (**Q**) or GSC 7-11 (**R**) cells were lysed and total RNA extracted and quantitative real-time PCR analysis was carried out using TaqMan gene expression assays. The graph depicts fold changes in ETV1 expression relative to the control GSCs. Data represent mean ± s.e.m. of three independent experiments performed in triplicate. *P<0.05 Student's *t*-test compared with control. (**S**) Representative immunohistochemistry images using anti-CIC

or anti-PJA1 antibodies of sections obtained from brains of intracranial U87-pGIPZ controls or U87-shPJA1-2 xenograft mice. Scale bar, 50 μm. The immunoblot data are representative of at least three separate experiments.

Supplementary Figure 5

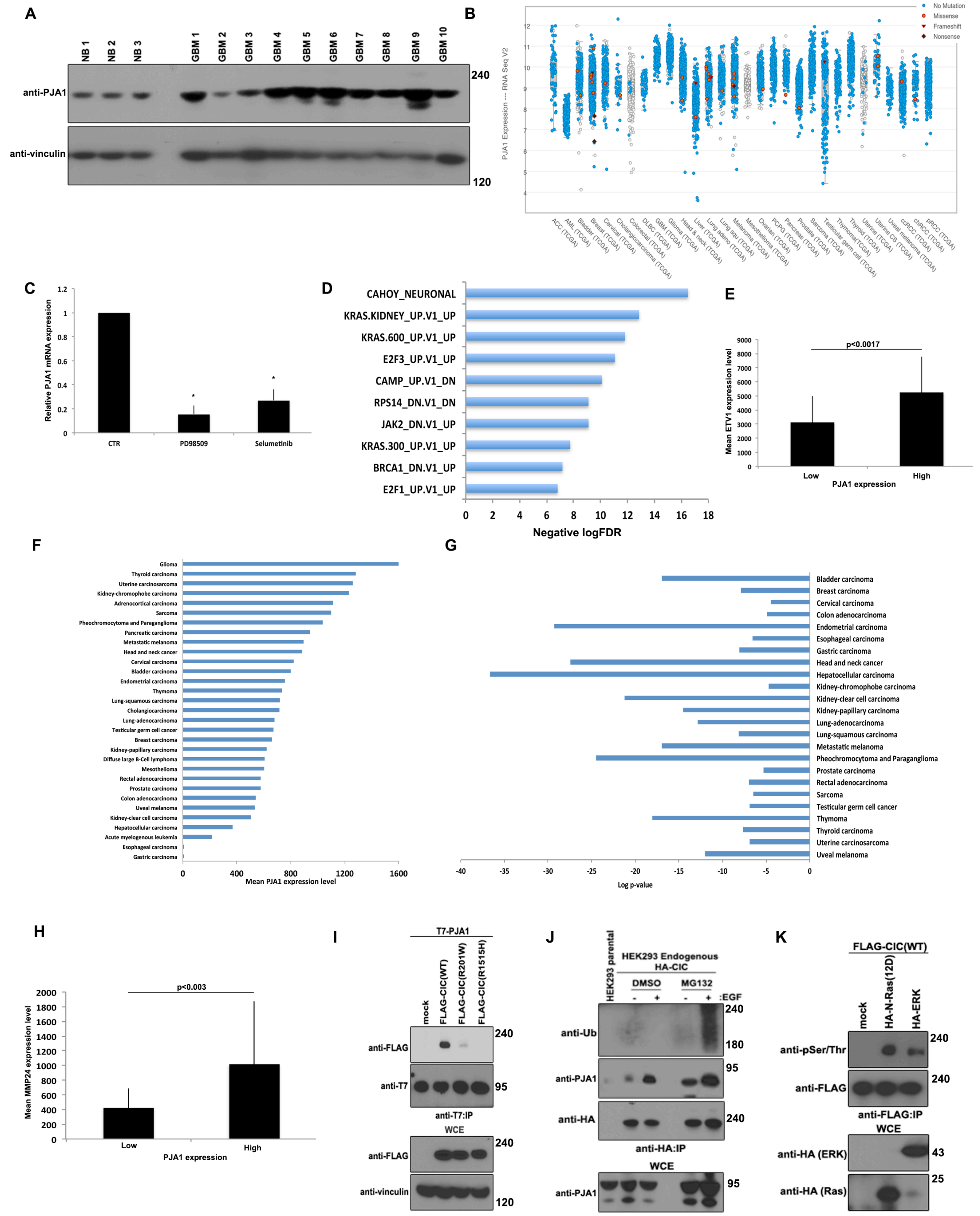

Supplementary Figure 5

PJA1 expression in human tumors, pathway analysis and PJA1-CIC interaction.

(**A**) Human operative GBM samples or normal derived brains (NB) were lysed and immunoblotted with indicated antibodies. (**B**) PJA1 mRNA expression profile across different tumor types according to the CBioPortal database. (**C**) U87 cell treated with or without DMSO, PD98509 or selumetinib for 24 hours were lysed and total RNA extracted and quantitative real-time PCR analysis was carried out using TaqMan gene expression assays. The graph depicts fold changes in PJA1 expression relative to the DMSO control. Data represent mean ± s.e.m. of three independent experiments performed in triplicate.*$P<0.05$ Student's *t*-test compared with DMSO control. (**D**) Oncogenic signaling pathways identified from gene set enrichment analysis (GSEA) of concordant genes up-regulated in the PJA1-high-expressing subsets of both IDH wild type GBMs and in IDH wild type lower-grade glioma. (**E**) ETV1 mRNA expression levels in IDH wild type GBMs in relation to PJA1 levels. ETV1 expression levels are plotted in for the bottom 25 versus top 25 PJA1-expressing GBMs in TCGA RNAseq level 3 data (total n=140). (**F**) Pan-TCGA comparison of mean PJA1 levels by tumor type. (**G**) Functional annotation analyses of genes correlated with PJA1 expression by tumor type in TCGA data. For each tumor type, a Pearson correlation analysis was performed to calculate the correlation of each gene with PJA1 expression. Using a p-value cutoff of <0.01, the top 500 genes from each tumor type were identified and subject to functional annotation using the online DAVID tool. The log (base 10) p-values for the recurring keyword "phosphoprotein" that was consistently significant in these datasets is shown. (**H**) Mean PJA1 expression level in CIC mutant versus CIC wild type IDH mutant

1p/19q co-deleted low grade gliomas (LGG) from TCGA. (**I**) MMP24 mRNA expression levels in IDH wild type GBMs in relation to PJA1 levels, plotted in a manner similar to Figure S5E. (**I**) HEK293 cells transfected with indicated plasmids were lysed, immunoprecipitated (IP) with anti-T7 antibody and immunoblotted with indicated antibodies. Whole cell extract (WCE). (**J**) HEK293 endogenously tagged HA-CIC or parental cells were pre-treated for 4 hours with or without DMSO or MG132 following EGF treatment were lysed and immunoprecipitated (IP) with anti-HA antibody and immunoblotted with indicated antibodies. Whole cell extract (WCE). (**K**) HEK293 cells transfected with indicated plasmids were lysed, immunoprecipitated (IP) with anti-FLAG antibody and immunoblotted with indicated antibodies. Whole cell extract (WCE). The immunoblot data are representative of at least three separate experiments.

Supplementary Figure 6

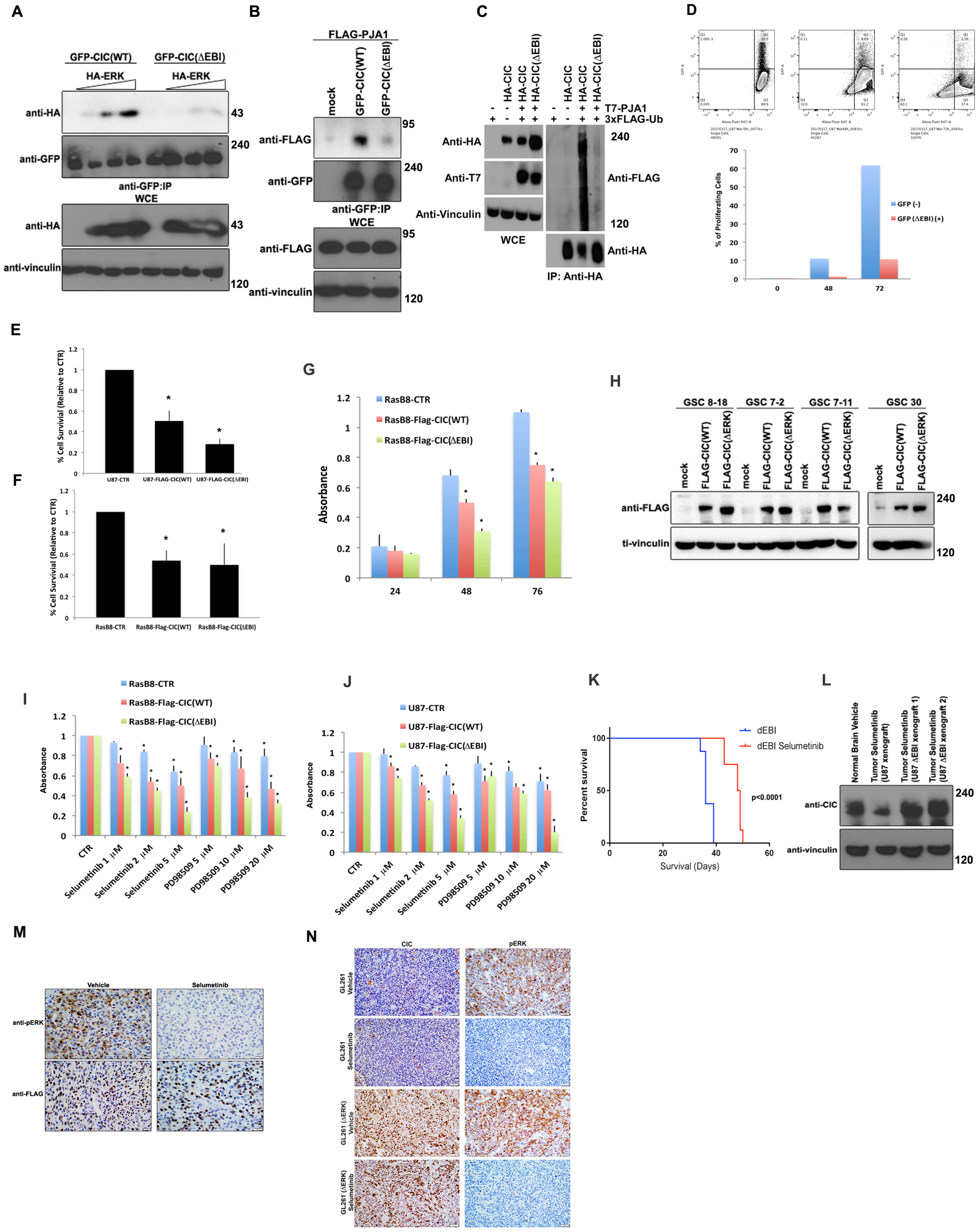

Supplementary Figure 6

Ubiquitylation assays and effect of CIC(ΔEBI) on proliferation. (**A**) and (**B**) HEK293 cells transfected with indicated plasmids were lysed and immunoprecipitated (IP) with anti-GFP antibody and immunoblotted with indicated antibodies. Whole cell extract (WCE). (**C**) HEK293 cells transfected with indicated plasmids were pre-treated with MG132 for 4 hours prior to 30 minute EGF treatment and were lysed and a denaturing immunoprecipitation (IP) using anti-HA antibody was performed followed by immunoblotting with indicated antibodies. Ubiquitin (UB); Whole cell extract (WCE). (**D**) U87 transfected with GFP-CIC(ΔEBI) labeled with the eFluor 670 proliferation dye were analyzed by flow cytometry at indicated time points. Graphs depict percentage of proliferating GFP-CIC(ΔEBI) versus GFP negative cells within the same experimental plate. Data are representative of at least three independent experiments. Equal number of either (**E**) U87 or (**F**) RasB8 empty plasmid control, or stably expressing FLAG-CIC or Flag-CIC(ΔEBI) cells were plated at the same density and trypan blue exclusion assay was conducted. Data represent mean ± s.e.m. of three independent experiments performed in octuplet. *$P<0.05$ Student's *t*-test compared with control (CTR). (**G**) Equal number RasB8 empty plasmid control, stably expressing FLAG-CIC or FLAG-CIC(ΔEBI) cells were plated and at indicated time points alamar blue assay was conducted. Data represent mean ± s.e.m. of three independent experiments performed in octuplet. *$P<0.05$ Student's *t*-test compared with control (CTR) within each cell line. (**H**) GSC 8-18, 7-2, 7-11 and 30 empty plasmid control, stably expressing FLAG-CIC or FLAG-CIC(ΔEBI) cells were lysed and immunoblotted with indicated antibodies. Equal number of (**I**) RasB8 or (**J**) U87 empty plasmid control, stably expressing FLAG-CIC or

FLAG-CIC(ΔEBI) cells were treated with or without DMSO control, PD98509 or selumetinib at indicated concentrations, and alamar blue assay conducted. Data represent mean ± s.e.m. of three independent experiments performed in octuplet. *$P<0.05$ Student's *t*-test compared with DMSO control (CTR) within each cell line. (**K**) Kaplan-Meier survival curves of mice with intracranial implantation of U87-CIC(ΔEBI) treated with vehicle or selumetinib. Log-Rank statistics were performed on the two groups. Eight-week-old mice were used with $n = 7$ per group. (**L**) Unaffected normal brain obtained from vehicle treated mice or tumor obtained from intracranial U87 xenograft mice treated with selumetinib or from two different U87-CIC(ΔEBI) xenograft mice treated with selumetinib were lysed and protein lysates were immunoblotted with indicated antibodies. (**M**) Representative immunohistochemistry images using anti-CIC or anti-pERK antibody of sections obtained from brains of intracranial U87-CIC(ΔEBI) xenograft mice treated with vehicle or selumetinib. (**N**) Representative images of immunohistochemistry using anti-CIC or anti-pERK antibody of sections obtained from brains of GL261 control or GL261-FLAG-CIC(ΔEBI) intracranial xenograft mice that were treated with either vehicle or selumetinib, beginning at 7 days post injections. Scale bar, 50 μm. The immunoblot data are representative of at least three separate experiments.

UNCROPPED BLOTS

Figure 1A

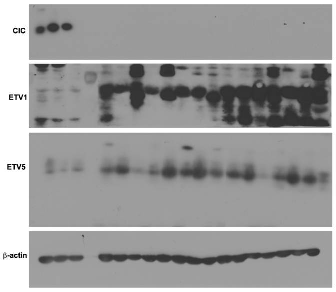

Figure 1C

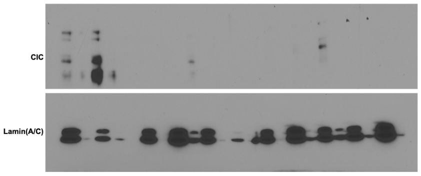

Figure 1G

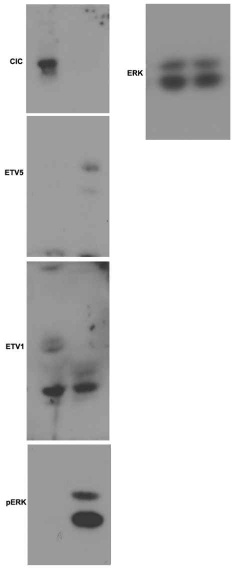

Figure 1E

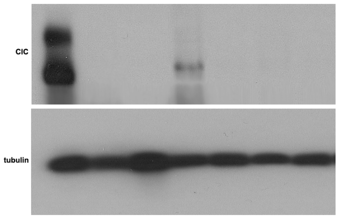

Figure 1D

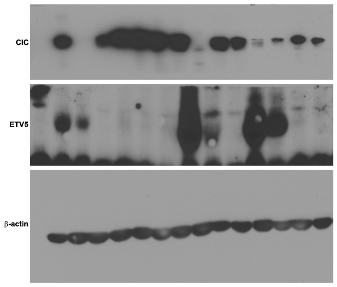

Figure 1H

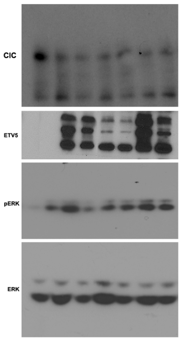

Figure 1L

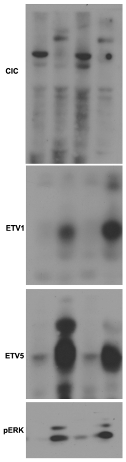

Figure 1M

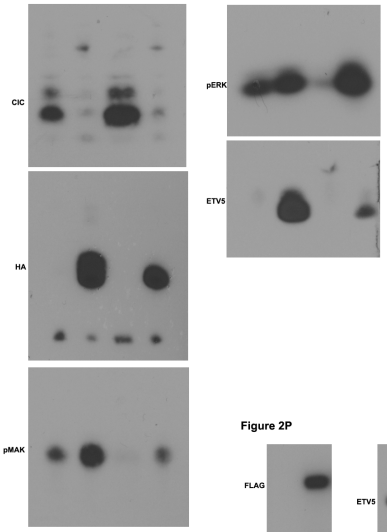

Figure 2A

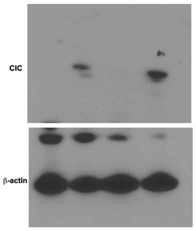

Figure 2B

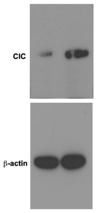

Figure 2D

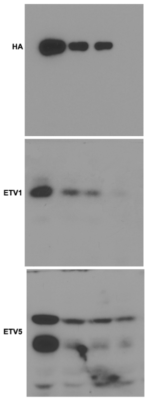

Figure 2P

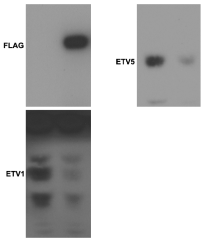

Figure 2H

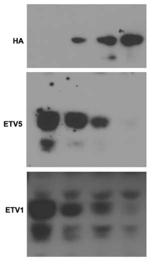

Figure 2M

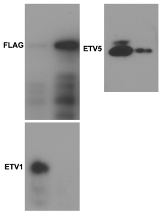

Figure 3A

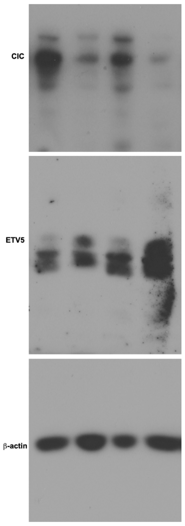

Figure 3E

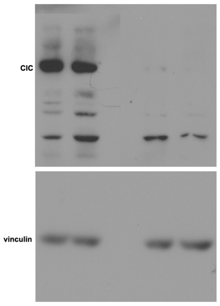

Figure 3I

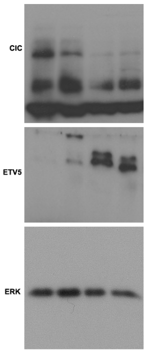

Figure 3L

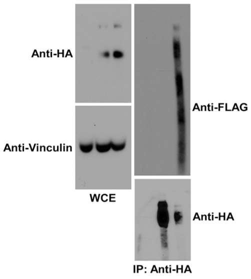

Figure 3M

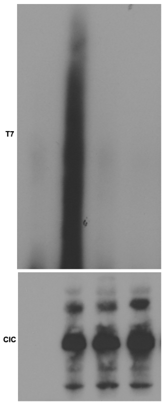

Figure 3N

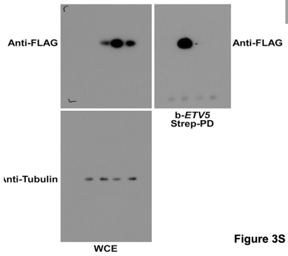

Figure 3R

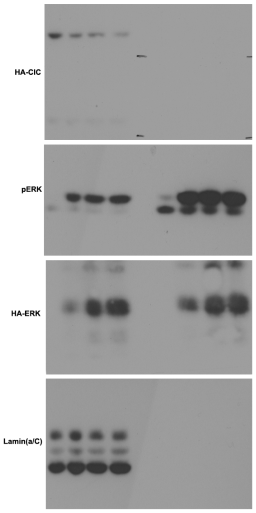

Figure 3Q

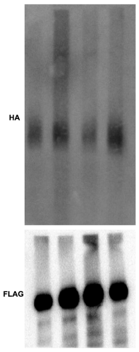

Figure 3S

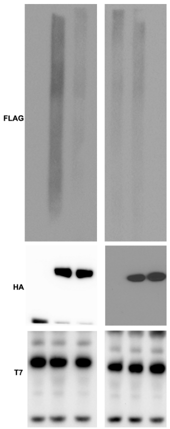

Figure 4A

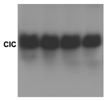

Figure 4C

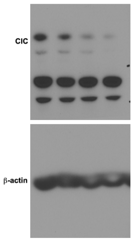

Figure 4B

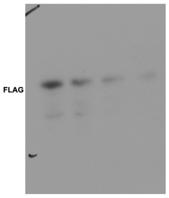

Figure 4J

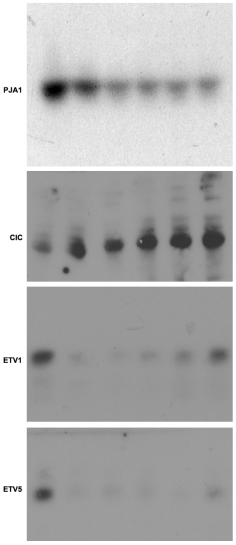

Figure 4F

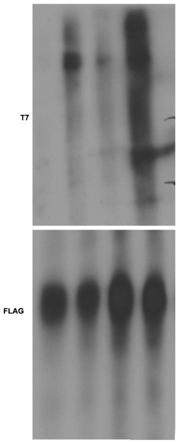

Figure 4G

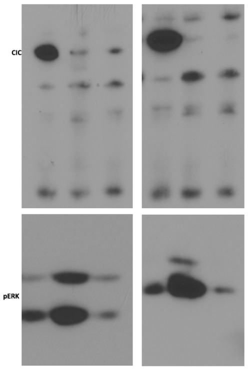

Figure 4D

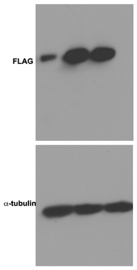

Figure 4E

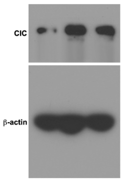

Figure 4L

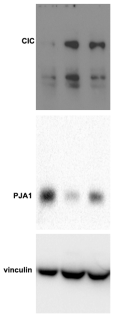

Figure 4N

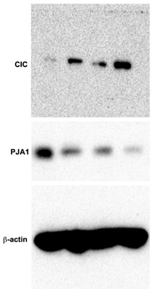

Figure 4P

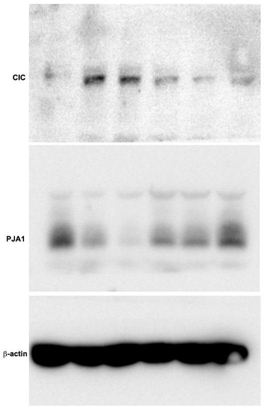

Figure 4R

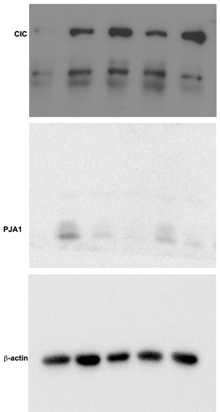

Figure 4U

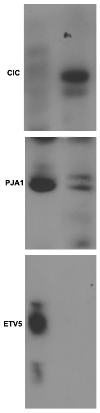

Figure 5A

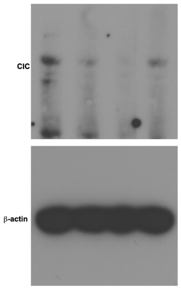

Figure 5D

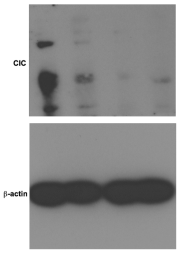

Figure 5G

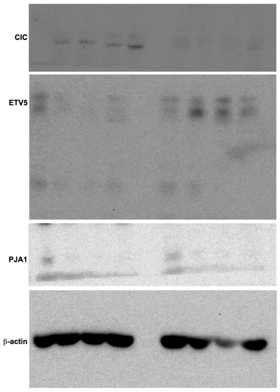

Figure 5I

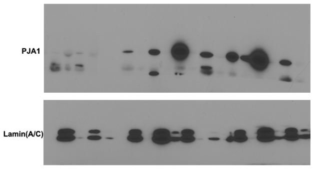

Figure 5K

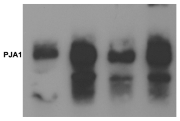

Figure 5M

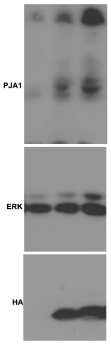

Figure 5O

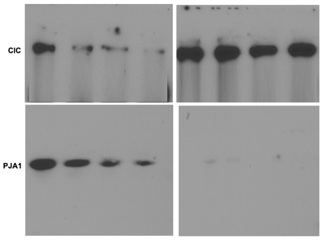

Figure 5P

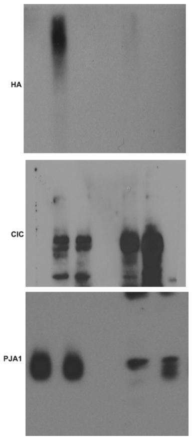

Figure 5L

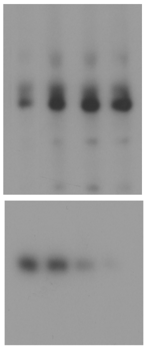

Figure 5Q

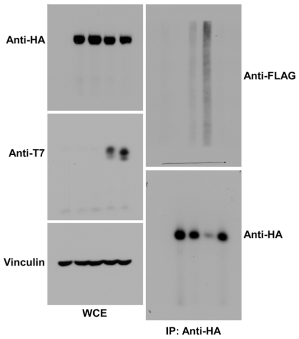

Figure 5R

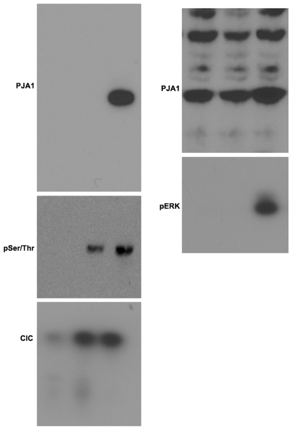

Figure 5S

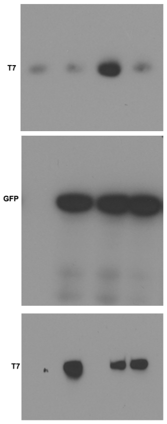

Figure 5T

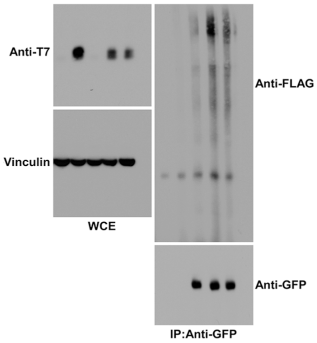

Figure 6A

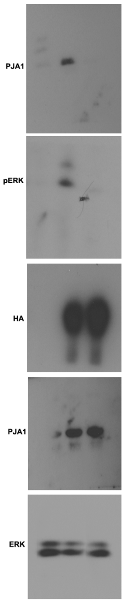

Figure 6B

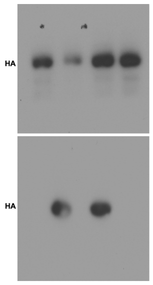

Figure 6D

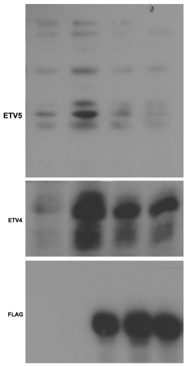

Figure 6E

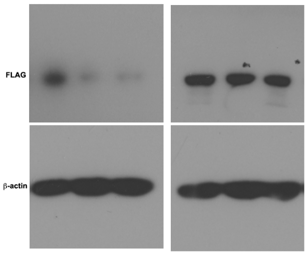

Figure 6F

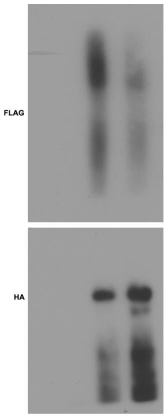

Figure 6G

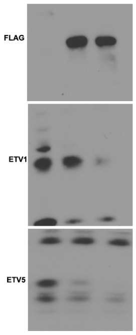

Supplement: Supplementary file 1 — Supplementary Information [file 41467_2018_8087_MOESM1_ESM.pdf]
