# Supplementary material for: CIC protein instability contributes to tumorigenesis in glioblastoma
Source: Nat Commun. 2019 Feb 8;10:661. doi: 10.1038/s41467-018-08087-9 (PMC6368580; doi:10.1038/s41467-018-08087-9)
Supplement: Supplementary file 2 — Reporting Summary [file 41467_2018_8087_MOESM2_ESM.pdf]

nature research

Corresponding author(s): *Double-blind peer review submissions: write DBPR and your manuscript number here instead of author names.*

Reporting Summary

Nature Research wishes to improve the reproducibility of the work that we publish. This form provides structure for consistency and transparency in reporting. For further information on Nature Research policies, see Authors & Referees and the Editorial Policy Checklist.

Statistical parameters

When statistical analyses are reported, confirm that the following items are present in the relevant location (e.g. figure legend, table legend, main text, or Methods section).

n/a	Confirmed	
☐	☒	The exact sample size (*n*) for each experimental group/condition, given as a discrete number and unit of measurement
☐	☒	An indication of whether measurements were taken from distinct samples or whether the same sample was measured repeatedly
☐	☒	The statistical test(s) used AND whether they are one- or two-sided<br>*Only common tests should be described solely by name; describe more complex techniques in the Methods section.*
☒	☐	A description of all covariates tested
☒	☐	A description of any assumptions or corrections, such as tests of normality and adjustment for multiple comparisons
☐	☒	A full description of the statistics including central tendency (e.g. means) or other basic estimates (e.g. regression coefficient) AND variation (e.g. standard deviation) or associated estimates of uncertainty (e.g. confidence intervals)
☐	☒	For null hypothesis testing, the test statistic (e.g. *F*, *t*, *r*) with confidence intervals, effect sizes, degrees of freedom and *P* value noted<br>*Give P values as exact values whenever suitable.*
☒	☐	For Bayesian analysis, information on the choice of priors and Markov chain Monte Carlo settings
☒	☐	For hierarchical and complex designs, identification of the appropriate level for tests and full reporting of outcomes
☒	☐	Estimates of effect sizes (e.g. Cohen's *d*, Pearson's *r*), indicating how they were calculated
☐	☒	Clearly defined error bars<br>*State explicitly what error bars represent (e.g. SD, SE, CI)*

Our web collection on statistics for biologists may be useful.

Software and code

Policy information about availability of computer code

Data collection	No software was used
Data analysis	No software used

For manuscripts utilizing custom algorithms or software that are central to the research but not yet described in published literature, software must be made available to editors/reviewers upon request. We strongly encourage code deposition in a community repository (e.g. GitHub). See the Nature Research guidelines for submitting code & software for further information.

Data

Policy information about availability of data

All manuscripts must include a data availability statement. This statement should provide the following information, where applicable:

- Accession codes, unique identifiers, or web links for publicly available datasets
- A list of figures that have associated raw data
- A description of any restrictions on data availability

The data supporting the findings of this study are available from the corresponding authors upon reasonable request.
The data that support the findings of this study are available from the corresponding
author upon reasonable request.

The data that support the findings of this study are available from the corresponding author upon reasonable request.

Field-specific reporting

Please select the best fit for your research. If you are not sure, read the appropriate sections before making your selection.

☒ Life sciences ☐ Behavioural & social sciences ☐ Ecological, evolutionary & environmental sciences

For a reference copy of the document with all sections, see nature.com/authors/policies/ReportingSummary-flat.pdf

Life sciences study design

All studies must disclose on these points even when the disclosure is negative.

Sample size	For mouse studies at a minimum 7 mice were used per experimental arm in line with molecular biology studies that rely on mice to reproduce novel cellular findings and ensure relevant statistical calculations could be performed
Data exclusions	None
Replication	All RT-PCR and CHIP studies were performed using three technical replicates three independent times. Cell proliferation assays were performed using 8 technical replicates three independent times.
Randomization	Random
Blinding	Blinding

Reporting for specific materials, systems and methods

Materials & experimental systems

n/a	Involved in the study	
☒	☐	Unique biological materials
☐	☒	Antibodies
☐	☒	Eukaryotic cell lines
☒	☐	Palaeontology
☐	☒	Animals and other organisms
☒	☐	Human research participants

Methods

n/a	Involved in the study	
☒	☐	ChIP-seq
☐	☒	Flow cytometry
☒	☐	MRI-based neuroimaging

Antibodies

Antibodies used	The following antibodies were obtained from Cell Signaling Technologies: HA (C29F4) (1:6,000), Lamin(A/C) (2032), pERK (4370), ubiquitin (3933), PJA2 (40180), and β-TrCP (D13F10), β-actin (8H10D10) (1:20,000), myc tag (71D10) and α-tubulin (2144) (1:5,000). pERK (sc-7383 and sc-16982-R), PJA1 (sc-517068) and GFP (sc-9996) (1:6,000) were obtained from Santa Cruz Biotechnology. Ki67 was obtained from Dako. Phospho Ser/Thr (ab17464), T7 tag (ab9138), capicua (ab123822), ETV1 (ab81086), Renilla (ab187338), and myc protein (ab3207) were obtained from Abcam. The following antibodies were purchased from Millipore T7 tag (69522), capicua (ABN446) and capicua (MABN449). FLAG-M2 (F1804), β-actin (A5316) (1:10,000), vinculin (V9264) (1:30,000), ETV5 (WH0002119M2) and polyclonal ERK (M5670) antibodies (1:5,000) were obtained from Sigma. PJA1 (MBS153701) was purchased from MyBioSource.com. All antibodies were utilized at a 1:1,000 dilution unless otherwise specified.
Validation	All antibodies used in the study are described on their respective manufacturers website and we validated each antibody detected the correct protein.

Eukaryotic cell lines

Policy information about cell lines

Cell line source(s)	HEK293A, HEK293T, U87, U251, U373, U118, A172, T98G and GL261 were obtained from American Type Culture Collection. NHA, U87-vIII cell line, an EGFRvIII expression derivative of U87, RasB8 cells were generated from Guha lab see reference 27. Six GSC cultures (GSC 8-18, GSC 7-2, GSC 7-11, GSC 28, GSC 267 and GSC 30) were derived from freshly operated tumor samples from GBM patients at the University of Texas MD Anderson Cancer Center as per guidelines set by institutional review board guidelines. NSCs were isolated and cultivated as previously described with slight modifications52. Briefly, 1-day old CIC flox x Rosa CreERT2 (BL6/N-Cictm1a(KOMP)Wtsi Gt(ROSA)26Sortm1(cre/ERT2)Tyj / Avd) mice were sacrificed and the whole brain was dissected out. The tissue was digested using Dispase, DNAse and Papain, washed and cultured in Neurobasal Medium (Invitrogen) supplemented with 2% B27 (Invitrogen), 2 mM Glutamax (Invitrogen) and the growth factors 20 ng/ml EGF (mouse recombinant; PeproTech) and 20 ng/ml bFGF (mouse recombinant; PeproTech). After 7 days in culture neurospheres were harvested by centrifugation, dissociated mechanically and replated. Cells were passaged once to twice weekly. To knockout CIC in mNSC, 1 µM of (Z)-4-Hydroxytamoxifen was added twice.
Authentication	The cell lines were not authenticated however they behaved as anticipated. Importantly, results were validated in primary patient specimens and patient derived glioma stem cells
Mycoplasma contamination	All cell lines were negative for mycoplasm
Commonly misidentified lines (See ICLAC register)	U87s and U373 were used in the study. They were purchased from ATCC

Animals and other organisms

Policy information about studies involving animals; ARRIVE guidelines recommended for reporting animal research

Laboratory animals	Male 8 week old C57/BL6 and NOD/SCID mice were used
Wild animals	None
Field-collected samples	None

Flow Cytometry

Plots

Confirm that:

- ☒ The axis labels state the marker and fluorochrome used (e.g. CD4-FITC).
- ☒ The axis scales are clearly visible. Include numbers along axes only for bottom left plot of group (a 'group' is an analysis of identical markers).
- ☒ All plots are contour plots with outliers or pseudocolor plots.
- ☒ A numerical value for number of cells or percentage (with statistics) is provided.

Methodology

Sample preparation	Cell lines were prepared using the Cell Proliferation Dye eFluor 670 (eBioscience) as per manufacturer’s instruction
Instrument	Becton Dickinson LSR II SC
Software	FlowJo software analysis
Cell population abundance	Cell population abundance was determined by a licensed operator at the Hospital for Sick Children who conducted all Flow Cytometry experiments
Gating strategy	Appropriate staining controls were used as provided in the Cell Proliferation Dye eFluor 670 (eBioscience) according to manufacturer’s instructions

- ☐ Tick this box to confirm that a figure exemplifying the gating strategy is provided in the Supplementary Information.
